# Supplementary material for: Dephosphorylation of YB-1 is Required for Nuclear Localisation During G2 Phase of the Cell Cycle
Source: Cancers (Basel). 2020 Jan 29;12(2):315. doi: 10.3390/cancers12020315 (PMC7072210; doi:10.3390/cancers12020315)
Supplement: Supplementary file 1 [file cancers-12-00315-s001.zip › cancers-661288-v2-suppl/Supplementary Figures/cancers-661288-Figure S1-S8.docx]

Supplementary Materials: Dephosphorylation of YB-1 is Required for Nuclear Localisation During G_2_ Phase of the Cell Cycle

Sunali Mehta, Cushla McKinney, Michael Algie, Chandra S. Verma, Srinivasaraghavan Kannan, Rhodri Harfoot, Tara K. Bartolec, Puja Bhatia, Alistair J. Fisher, Maree L. Gould, Kim Parker, Anthony J. Cesare, Heather E. Cunliffe, Scott B. Cohen, Torsten Kleffmann, Antony W. Braithwaite and Adele G. Woolley


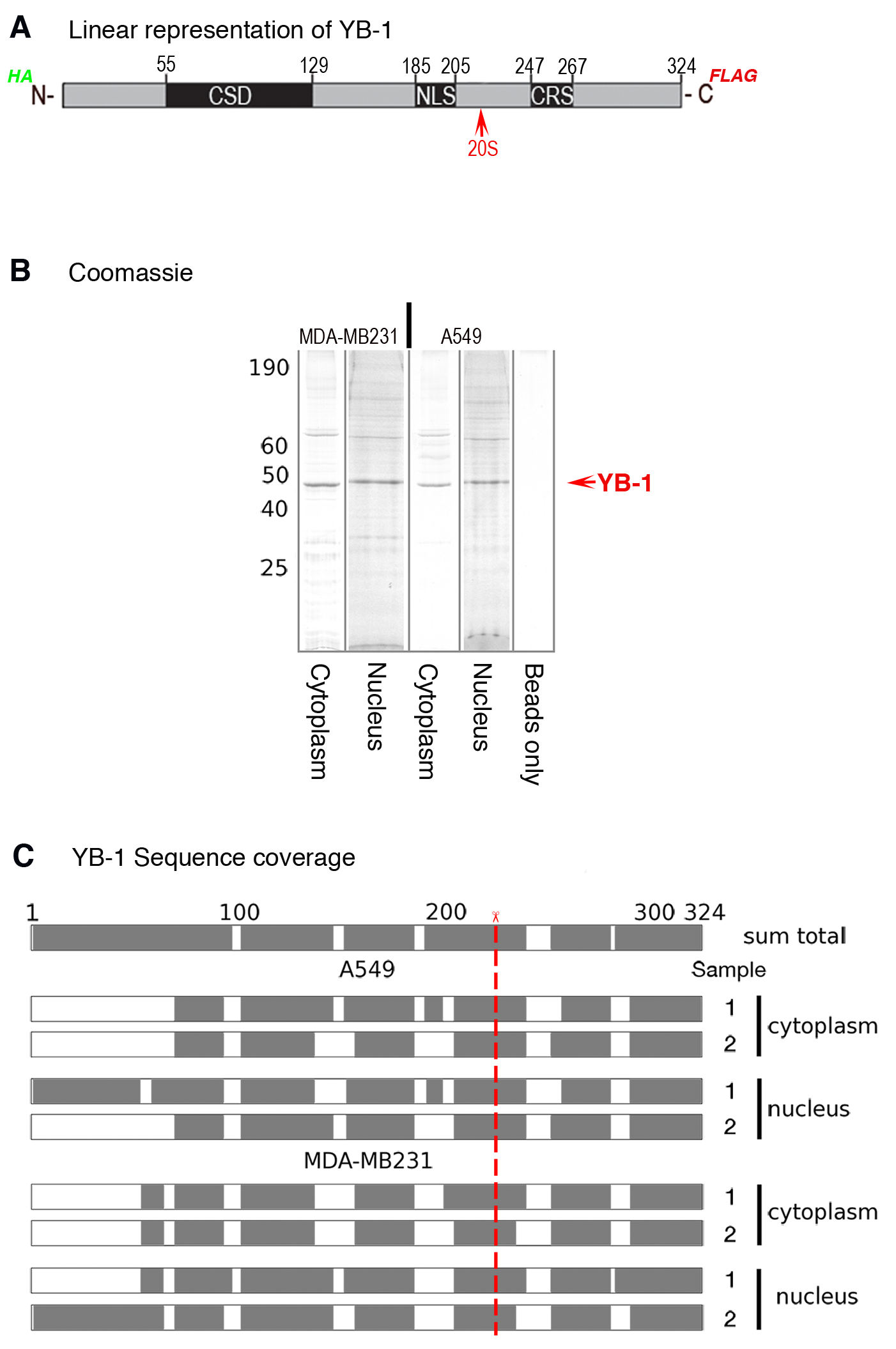


**Figure S1.** (**A**) **Linear representation of YB-1.** YB-1 contains a highly conserved cold shock domain (CSD); a nuclear localisation site (NLS) and a cytoplasmic retention site (CRS); 20S (arrowed in red) beneath the graphic denotes the site of proposed cleavage by the 20S Proteasome between amino acids 219/220. HA and FLAG tags are shown at the at the N- and C-terminal respectively. (**B**) **Representative coomassie stain of purified cytoplasmic and nuclear YB-1 from A549 and MDA- MB231 cells**. YB-1 is denoted with an arrow. (**C**) the sequence coverage from peptides for YB-1 identified by LC-MS/MS for two replicate samples (1 & 2) for each of the cell lines are shown. The dashed red line shows the proposed cleavage site (219/220).

**Figure S2.** (**A**) **Cell survival curves following treatment with either Doxorubicin (DOX) or Paclitaxel (PTX).** The IC50 value for each drug, whereby 50% of cells survive, is shown by the red dotted line. (**B**) **Immunoblots from cellular fractions of A549, H1299 and Saos-2 cells.** The left-hand blots show *^HA^*YB-1 (arrowed) in green; the middle blots YB-1 *^FLAG^* in red; the right-hand blots are a merge of both red and green labels. WCL denotes the whole cell (unfractionated); Nuc denotes the nuclear and cytoskeletal fragment; Cyto, the cytoplasmic fragment. The presence of histone (H3) is also shown under the left-hand blots. On each blot, the first set (WCL, Nuc, Cyto) was untreated with either doxorubicin (DOX) or paclitaxel (PTX); the second set was treated with PTX, the third set with DOX. Densitometries for these western blots is shown in Table S1-S3 (Supplementary Materials)


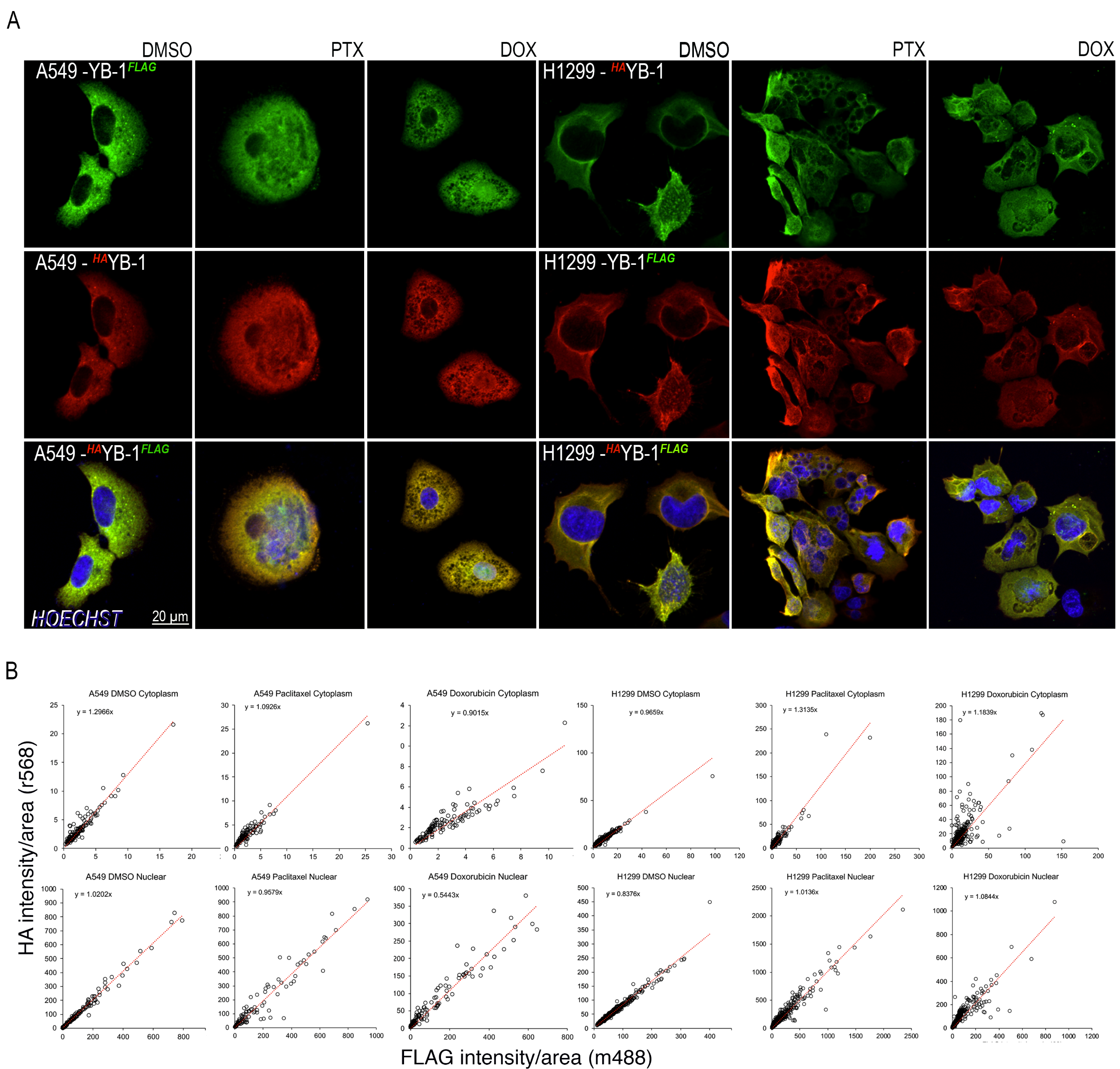


**Figure S3. Immunofluorescent labelling with alternative antibodies.** (**A**) shows the nuclear and cytoplasmic location of *^HA^*YB-1*^FLAG^* in A549 and H1299 cells treated with DOX or PTX and labelled with anti-mouse 488 and anti-rabbit 568 antibodies to FLAG and HA, respectively. Top row shows anti-FLAG (which detects the C-terminus of tagged *^HA^*YB-1*^FLAG^*, middle row shows anti-HA which detects the N-terminus of tagged *^HA^*YB-1*^FLAG^* and the bottom row shows the merged image. The nucleus is stained with Hoechst. (**B**) the fluorescence intensity/area for anti-HA and anti-FLAG using masks to differentiate the nucleus and the cytoplasmic compartments (as previously shown in Figure 1B) for > 100 cells in both cell lines. Each dot represents the intensity/area for anti-HA and anti-FLAG for each cell. The red dotted line represents the line of best fit. The slope of the line is indicated.


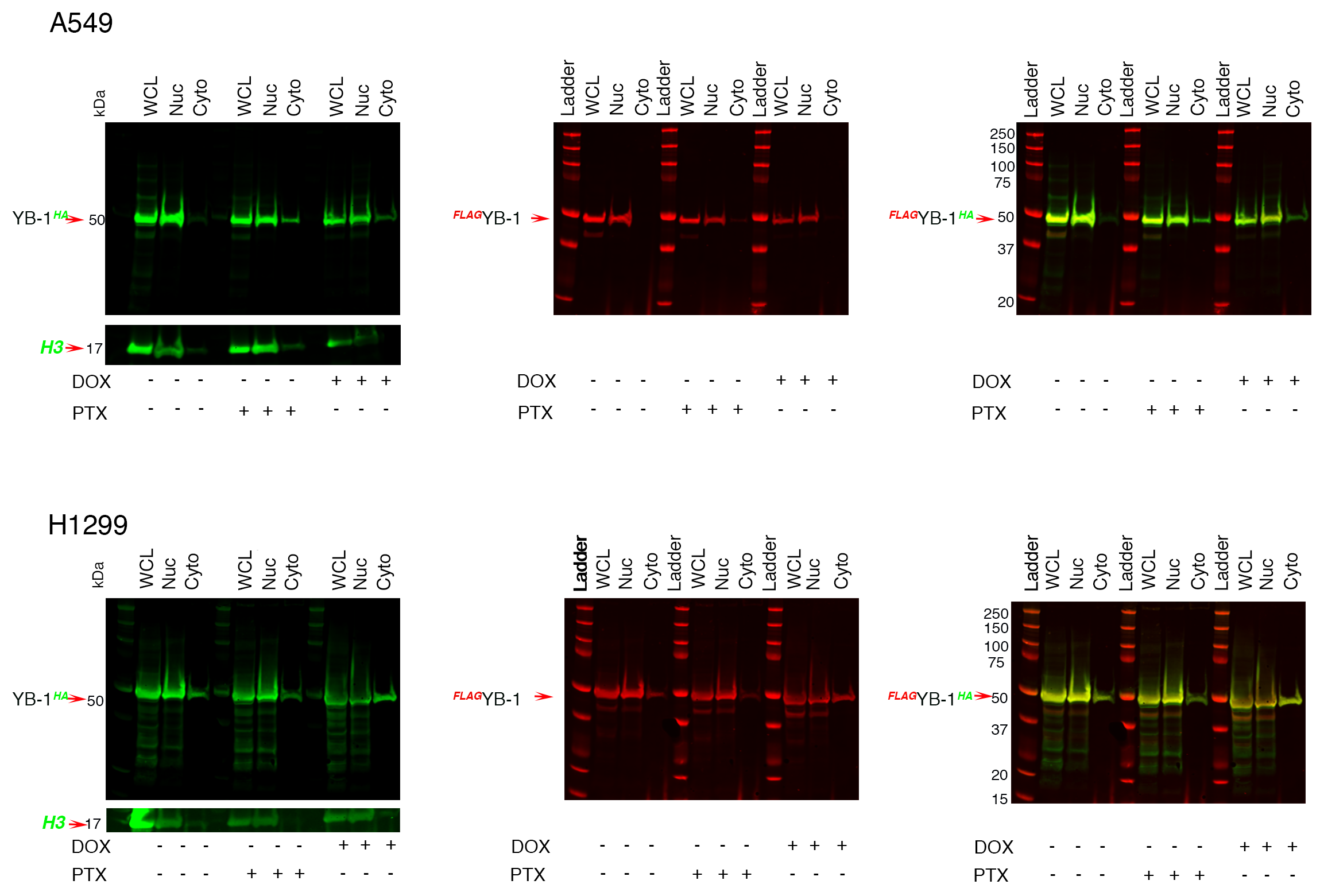


**Figure S4.** **Immunoblots from cellular fractions of A549 and H1299 following transfection with *^FLAG^*YB-1*^HA^* construct.** The left-hand blots show YB-1*^HA^* (arrowed) in green; the middle blots *^FLAG^*YB-1 in red; the right-hand blots are a merge of both red and green labels ^FLAG^YB-1*^HA^*. WCL denotes the whole cell (unfractionated); Nuc denotes the nuclear and cytoskeletal fragment; Cyto, the cytoplasmic fragment. The presence of histone (H3) is also shown under the left-hand blots. On each blot, the first set (WCL, Nuc, Cyto) was untreated with either doxorubicin (DOX) or paclitaxel (PTX); the second set was treated with PTX, the third set with DOX. Densitometries for these western blots is shown in Table S6 (Supplementary Materials)


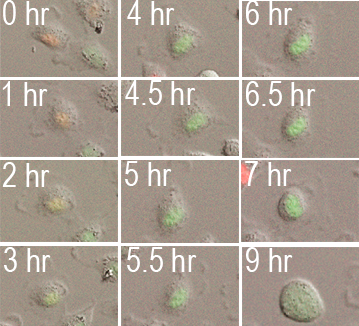


**Figure S5. Cell-cycle progression following release from double thymidine block.** Timepoints from 0-9 hours following release from double thymidine block in an A549 FUCCI cell. The cell S phase at 0 – 2 hr post release (yellow); S-G_2_ (green) from 3-6 hrs; G_2_ at 7 hrs and M at 9 hours post release of double thymidine block.


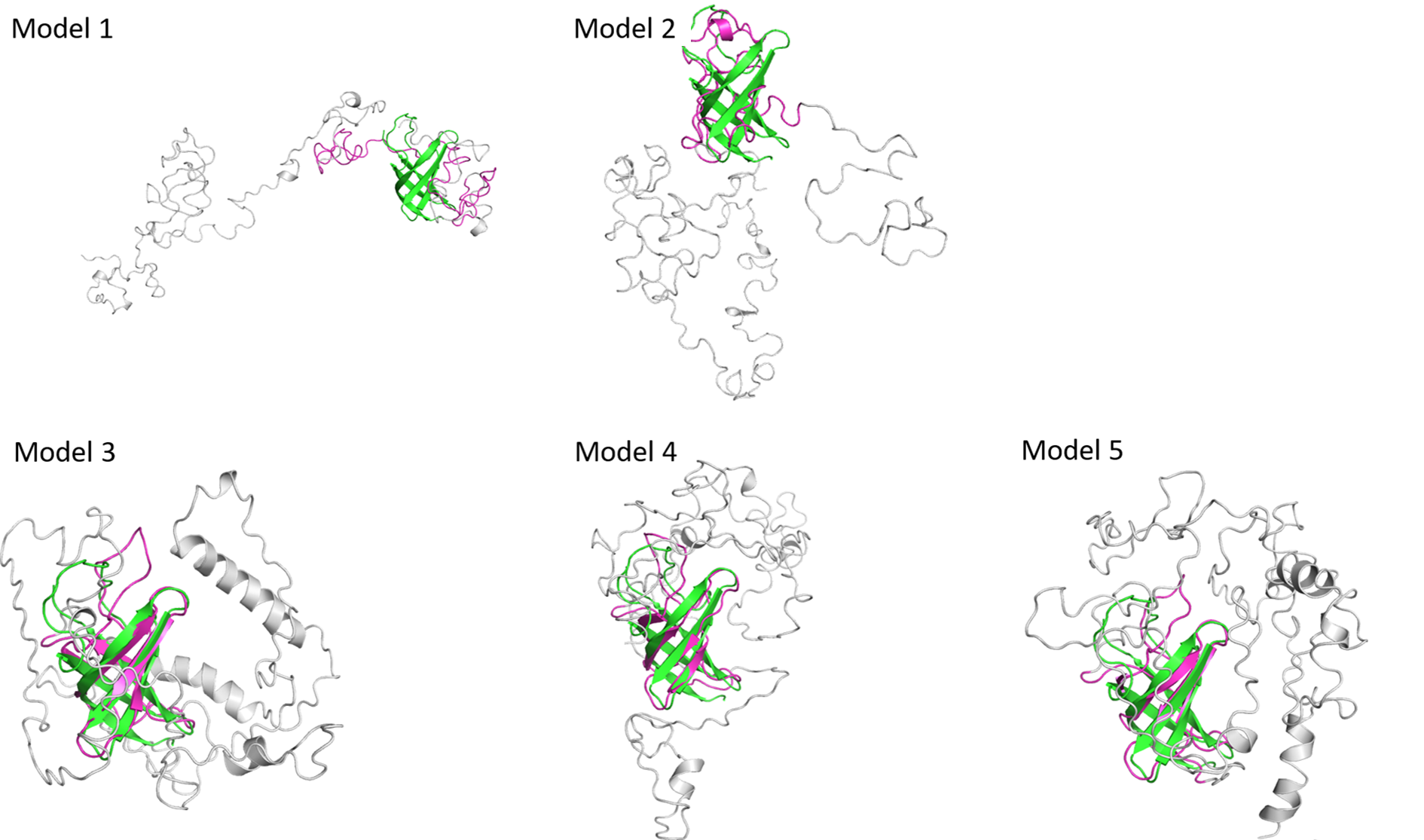


**Figure S6.** **Models generated by I-TASSER server.** Model 3 was chosen due to the fact that the predicted cold shock domain was close to the known NMR structure of YB-1. The green coloured structures are the NMR structures (PDB 1H95); while the modelled structures (from I-TASSER) are shown in grey except for the Y-BOX cold shock domain, which is shown in magenta.


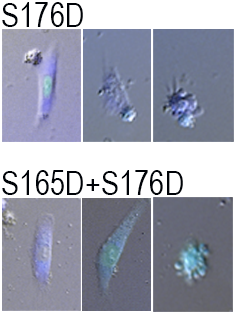


**Figure S7.** **Live-cell images** **cells with a serine (S) to aspartic acid (D) mutation.** Cells have been transfected with an YB-1*^EBFP2^* construct (blue). YB-1 is retained in the cytoplasm of cells with either a single mutation at serine 176 or a double mutation at S165 and S176. These cells undergo apoptosis as they begin to enter M phase


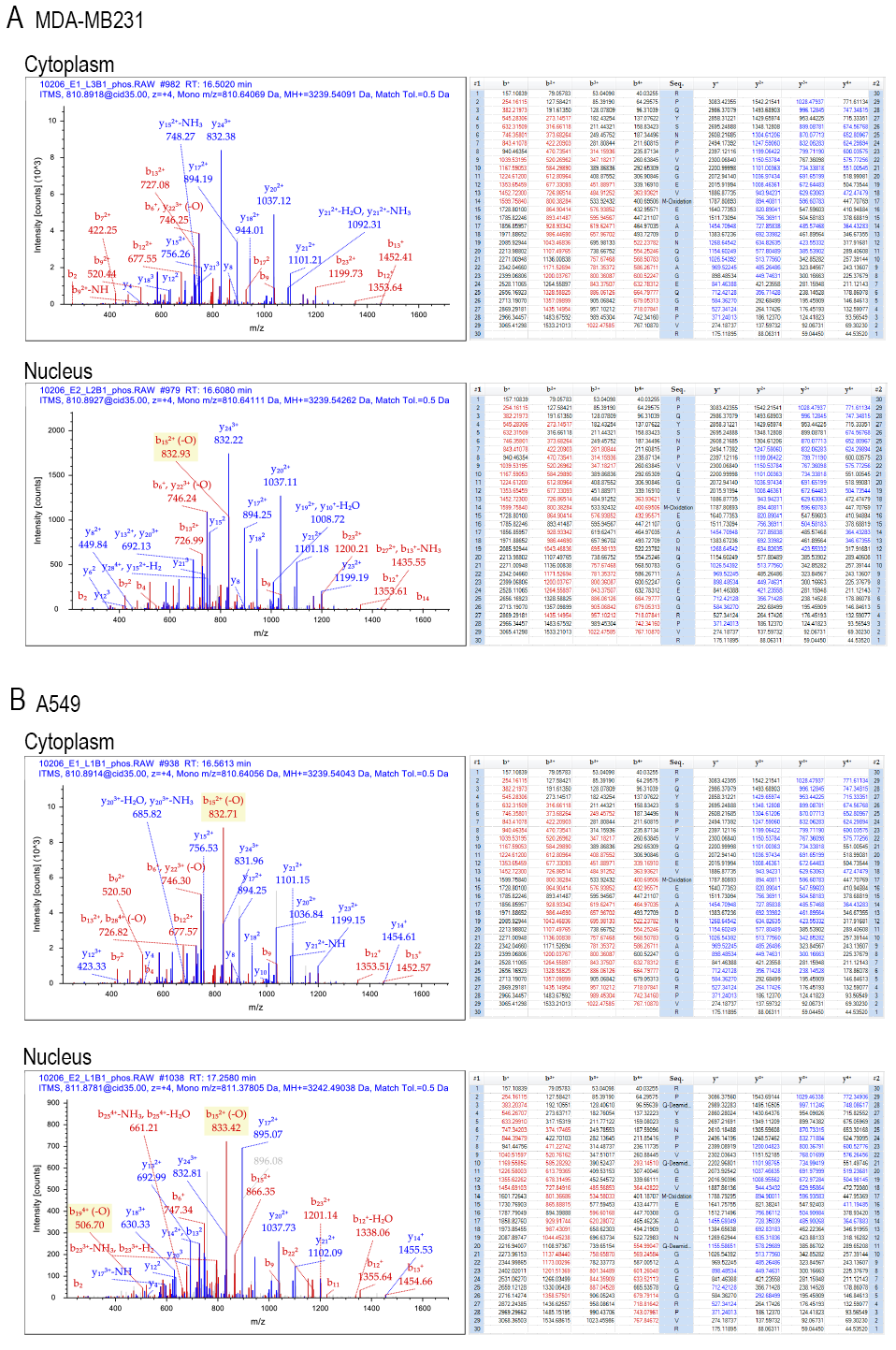


**Figure S8. Peptide spectrum matches for a YB-1 peptide that spans a proposed 20S cleavage site found in the cytoplasm and nucleus of MDA-MB231 and A549 cells.** Representative spectra for RPQYSNPPVQGEVMEGADNQGAGEQGRPVR, a YB-1 peptide that was detected in the cytoplasm and nucleus of MDA-MB231 (**A**) and A549 (**B**) cells. The tables adjacent to the spectra highlight the product ions that were identified in each spectrum and contributed to the identification of the peptide. **Table S5** provides a list of all YB-1 peptides confidently identified in the YB-1 band.
