# Supplementary material for: Dephosphorylation of YB-1 is Required for Nuclear Localisation During G2 Phase of the Cell Cycle
Source: Cancers (Basel). 2020 Jan 29;12(2):315. doi: 10.3390/cancers12020315 (PMC7072210; doi:10.3390/cancers12020315)
Supplement: Supplementary file 1 [file cancers-12-00315-s001.zip › cancers-661288-v2-suppl/Supplementary Tables/Supplementary Tables of intensity S1-S4.docx]

Table S1: Signal intensity for ^HA^YB-1, YB-1^FLAG^ and H3 in A549 whole cell lysates (WCL), nuclear (Nuc) and cytoplasmic (Cyto) fractions.

| **Box Number** | **Sample Description** | **Signal Detected** | **Signal** | **Total** | **Area** | **Channel** |
| --- | --- | --- | --- | --- | --- | --- |
| **^HA^YB-1** | | | | | | |
| 1 | Background | Background | 0 | 149.7988 | 189 | 800 |
| 21 | WCL - DMSO | ^HA^YB-1 | 9460.472708 | 9942.365 | 608 | 800 |
| 22 | Nuc - DMSO | ^HA^YB-1 | 4548.032278 | 5029.925 | 608 | 800 |
| 23 | Cyto - DMSO | ^HA^YB-1 | 2970.521536 | 3452.414 | 608 | 800 |
| 24 | WCL - PTX | ^HA^YB-1 | 5720.943411 | 6202.836 | 608 | 800 |
| 25 | Nuc - PTX | ^HA^YB-1 | 6641.507376 | 7123.4 | 608 | 800 |
| 26 | Cyto - PTX | ^HA^YB-1 | 926.6982939 | 1408.591 | 608 | 800 |
| 27 | WCL - DOX | ^HA^YB-1 | 7679.743704 | 8161.636 | 608 | 800 |
| 28 | Nuc - DOX | ^HA^YB-1 | 4563.169485 | 5045.062 | 608 | 800 |
| 29 | Cyto - DOX | ^HA^YB-1 | 2135.091849 | 2616.984 | 608 | 800 |
| **YB-1^FLAG^** | | | | | | |
| 2 | Background | Background | 0 | 807.1699 | 189 | 700 |
| 30 | WCL - DMSO | YB-1^FLAG^ | 5364.161086 | 6961.418 | 374 | 700 |
| 31 | Nuc - DMSO | YB-1^FLAG^ | 3766.067336 | 5363.324 | 374 | 700 |
| 32 | Cyto - DMSO | YB-1^FLAG^ | 2380.707961 | 3977.965 | 374 | 700 |
| 33 | WCL - PTX | YB-1^FLAG^ | 3492.666946 | 5089.924 | 374 | 700 |
| 34 | Nuc - PTX | YB-1^FLAG^ | 3716.786086 | 5314.043 | 374 | 700 |
| 35 | Cyto - PTX | YB-1^FLAG^ | 202.2528832 | 1799.51 | 374 | 700 |
| 36 | WCL - DOX | YB-1^FLAG^ | 5440.274368 | 7037.531 | 374 | 700 |
| 37 | Nuc - DOX | YB-1^FLAG^ | 3584.575149 | 5181.832 | 374 | 700 |
| 38 | Cyto - DOX | YB-1^FLAG^ | 1111.721633 | 2708.979 | 374 | 700 |
| **H3** | | | | | | |
| 1 | Background | Background | 0 | 193.8875 | 408 | 800 |
| 2 | WCL - DMSO | H3 | 8021.059935 | 8430.22 | 861 | 800 |
| 3 | Nuc - DMSO | H3 | 6942.834105 | 7351.994 | 861 | 800 |
| 4 | Cyto - DMSO | H3 | 719.1187726 | 1128.278 | 861 | 800 |
| 5 | WCL - PTX | H3 | 534.560423 | 943.72 | 861 | 800 |
| 6 | Nuc - PTX | H3 | 382.6590558 | 791.8186 | 861 | 800 |
| 7 | Cyto - PTX | H3 | 84.30846988 | 493.468 | 861 | 800 |
| 8 | WCL - DOX | H3 | 696.94763 | 1106.107 | 861 | 800 |
| 9 | Nuc - DOX | H3 | 234.7994367 | 643.959 | 861 | 800 |
| 10 | Cyto - DOX | H3 | 72.96252262 | 482.1221 | 861 | 800 |

Table S2: Signal intensity for ^HA^YB-1, YB-1^FLAG^ and H3 in H1299 whole cell lysates (WCL), nuclear (Nuc) and cytoplasmic (Cyto) fractions.

| **Box Number** | **Sample Description** | **Signal Detected** | **Signal** | **Total** | **Area** | **Channel** |
| --- | --- | --- | --- | --- | --- | --- |
| **^HA^YB-1** | | | | | | |
| 1 | Background | Background | 0 | 587.4077 | 600 | 800 |
| 3 | WCL - DMSO | ^HA^YB-1 | 24776.89169 | 25247.8 | 481 | 800 |
| 12 | Nuc - DMSO | ^HA^YB-1 | 19925.63546 | 20396.54 | 481 | 800 |
| 13 | Cyto - DMSO | ^HA^YB-1 | 17819.86953 | 18290.78 | 481 | 800 |
| 14 | WCL - PTX | ^HA^YB-1 | 25881.09872 | 26352 | 481 | 800 |
| 15 | Nuc - PTX | ^HA^YB-1 | 30560.63632 | 31031.54 | 481 | 800 |
| 16 | Cyto - PTX | ^HA^YB-1 | 17713.535 | 18184.44 | 481 | 800 |
| 17 | WCL - DOX | ^HA^YB-1 | 16074.45614 | 16545.36 | 481 | 800 |
| 18 | Nuc - DOX | ^HA^YB-1 | 22166.31912 | 22637.22 | 481 | 800 |
| 19 | Cyto - DOX | ^HA^YB-1 | 13692.88449 | 14163.79 | 481 | 800 |
| **YB-1^FLAG^** | | | | | | |
| 2 | Background | Background | 0 | 4123.188 | 396 | 700 |
| 20 | WCL - DMSO | YB-1^FLAG^ | 16948.89631 | 23039.97 | 585 | 700 |
| 21 | Nuc - DMSO | YB-1^FLAG^ | 13828.85724 | 19919.93 | 585 | 700 |
| 22 | Cyto - DMSO | YB-1^FLAG^ | 12512.78693 | 18603.86 | 585 | 700 |
| 23 | WCL - PTX | YB-1^FLAG^ | 18342.06818 | 24433.14 | 585 | 700 |
| 24 | Nuc - PTX | YB-1^FLAG^ | 20299.16193 | 26390.23 | 585 | 700 |
| 25 | Cyto - PTX | YB-1^FLAG^ | 12872.40412 | 18963.48 | 585 | 700 |
| 26 | WCL - DOX | YB-1^FLAG^ | 17764.24006 | 23855.31 | 585 | 700 |
| 27 | Nuc - DOX | YB-1^FLAG^ | 13818.30256 | 19909.38 | 585 | 700 |
| - | Cyto - DOX | no image to quantify | | | | |
| **H3** | | | | | | |
| 1 | Background | Background | 0 | 288.3247 | 528 | 800 |
| 2 | WCL - DMSO | H3 | 10870.49969 | 11275.14 | 741 | 800 |
| 11 | Nuc - DMSO | H3 | 15063.68866 | 15468.33 | 741 | 800 |
| 12 | Cyto - DMSO | H3 | 1584.933286 | 1989.571 | 741 | 800 |
| 13 | WCL - PTX | H3 | 15666.86053 | 16071.5 | 741 | 800 |
| 14 | Nuc - PTX | H3 | 8236.240659 | 8640.878 | 741 | 800 |
| 15 | Cyto - PTX | H3 | 2079.317868 | 2483.955 | 741 | 800 |
| 16 | WCL - DOX | H3 | 9433.451596 | 9838.089 | 741 | 800 |
| 17 | Nuc - DOX | H3 | 7289.313657 | 7693.951 | 741 | 800 |
| 18 | Cyto - DOX | H3 | 735.5822116 | 1140.22 | 741 | 800 |

Table S3: Signal intensity for ^HA^YB-1, YB-1^FLAG^ and H3 in Saos-2 whole cell lysates (WCL), nuclear (Nuc) and cytoplasmic (Cyto) fractions.

| **Box Number** | **Sample Description** | **Signal Detected** | **Signal** | **Total** | **Area** | **Channel** |
| --- | --- | --- | --- | --- | --- | --- |
| **^HA^YB-1** | | | | | | |
| 1 | Background | Background | 0 | 2624.848 | 320 | 800 |
| 4 | WCL - DMSO | ^HA^YB-1 | 257830.291 | 261767.6 | 480 | 800 |
| 13 | Nuc - DMSO | ^HA^YB-1 | 239245.5488 | 243182.8 | 480 | 800 |
| 14 | Cyto - DMSO | ^HA^YB-1 | 84431.68164 | 88368.95 | 480 | 800 |
| 15 | WCL - PTX | ^HA^YB-1 | 212397.9941 | 216335.3 | 480 | 800 |
| 16 | Nuc - PTX | ^HA^YB-1 | 207197.8223 | 211135.1 | 480 | 800 |
| 17 | Cyto - PTX | ^HA^YB-1 | 57452.42383 | 61389.7 | 480 | 800 |
| 18 | WCL - DOX | ^HA^YB-1 | 147443.5566 | 151380.8 | 480 | 800 |
| 19 | Nuc - DOX | ^HA^YB-1 | 227918.5566 | 231855.8 | 480 | 800 |
| 20 | Cyto - DOX | ^HA^YB-1 | 114508.2129 | 118445.5 | 480 | 800 |
| **YB-1^FLAG^** | | | | | | |
| 3 | Background | Background | 0 | 5672.75 | 210 | 700 |
| 21 | WCL - DMSO | YB-1^FLAG^ | 543443.0938 | 560461.4 | 630 | 700 |
| 22 | Nuc - DMSO | YB-1^FLAG^ | 495999.0938 | 513017.3 | 630 | 700 |
| 23 | Cyto - DMSO | YB-1^FLAG^ | 141361.6875 | 158379.9 | 630 | 700 |
| 24 | WCL - PTX | YB-1^FLAG^ | 373766.6563 | 390784.9 | 630 | 700 |
| 25 | Nuc - PTX | YB-1^FLAG^ | 375074.6563 | 392092.9 | 630 | 700 |
| 26 | Cyto - PTX | YB-1^FLAG^ | 82071.21875 | 99089.47 | 630 | 700 |
| 27 | WCL - DOX | YB-1^FLAG^ | 245611.4063 | 262629.7 | 630 | 700 |
| 28 | Nuc - DOX | YB-1^FLAG^ | 411640.875 | 428659.1 | 630 | 700 |
| 29 | Cyto - DOX | YB-1^FLAG^ | 195706.0313 | 212724.3 | 630 | 700 |
| **H3** | | | | | | |
| 1 | Background | Background | 0 | 683.1904 | 324 | 800 |
| 2 | WCL - DMSO | H3 | 4291.900354 | 5177.518 | 420 | 800 |
| 3 | Nuc - DMSO | H3 | 6737.623011 | 7623.24 | 420 | 800 |
| 4 | Cyto - DMSO | H3 | 6856.197229 | 7741.814 | 420 | 800 |
| 5 | WCL - PTX | H3 | 39934.88278 | 40820.5 | 420 | 800 |
| 6 | Nuc - PTX | H3 | 68548.16403 | 69433.78 | 420 | 800 |
| 7 | Cyto - PTX | H3 | 8595.400354 | 9481.018 | 420 | 800 |
| 8 | WCL - DOX | H3 | 38495.00778 | 39380.63 | 420 | 800 |
| 9 | Nuc - DOX | H3 | 37925.79684 | 38811.41 | 420 | 800 |
| 10 | Cyto - DOX | H3 | 7753.306604 | 8638.924 | 420 | 800 |

Table S4: Signal intensity for total YB-1, pYB-1^S102^ and β-actin in A549 cells after release from double thymidine block over 24hours.

| **Box Number** | **Sample Description** | **Signal Detected** | **Signal** | **Total** | **Area** | **Channel** |
| --- | --- | --- | --- | --- | --- | --- |
| **YB-1 and β-actin** | | | | | | |
| 2 | Background | Background | 0 | 75.74097 | 161 | 800 |
| 17 | 4h | YB-1 | 1853.547385 | 2018.202 | 350 | 800 |
| 18 | 4h | YB-1 | 1723.209006 | 1887.863 | 350 | 800 |
| 19 | 6h | YB-1 | 1950.692404 | 2115.347 | 350 | 800 |
| 20 | 6h | YB-1 | 1865.26882 | 2029.923 | 350 | 800 |
| 21 | 7h | YB-1 | 1842.541037 | 2007.195 | 350 | 800 |
| 22 | 7h | YB-1 | 1605.785177 | 1770.439 | 350 | 800 |
| 23 | 8h | YB-1 | 2171.775412 | 2336.43 | 350 | 800 |
| 24 | 8h | YB-1 | 2201.676779 | 2366.331 | 350 | 800 |
| 25 | 11h | YB-1 | 940.5400603 | 1105.194 | 350 | 800 |
| 26 | 11h | YB-1 | 1336.988791 | 1501.643 | 350 | 800 |
| 27 | 24h | YB-1 | 3270.371115 | 3435.025 | 350 | 800 |
| 28 | 24h | YB-1 | 3354.930685 | 3519.585 | 350 | 800 |
| 29 | si-YB-1 | YB-1 | 375.0884001 | 539.7427 | 350 | 800 |
| 30 | si-Control | YB-1 | 1006.432256 | 1085.466 | 168 | 800 |
| 1 | Background | β-actin | 0 | 199.373 | 126 | 700 |
| 3 | 4h | β-actin | 6571.190647 | 7147.157 | 364 | 700 |
| 4 | 4h | β-actin | 4971.25803 | 5547.225 | 364 | 700 |
| 5 | 6h | β-actin | 6058.023655 | 6633.99 | 364 | 700 |
| 6 | 6h | β-actin | 4302.272678 | 4878.239 | 364 | 700 |
| 7 | 7h | β-actin | 3421.6926 | 3997.659 | 364 | 700 |
| 8 | 7h | β-actin | 4025.792209 | 4601.759 | 364 | 700 |
| 9 | 8h | β-actin | 4145.22092 | 4721.188 | 364 | 700 |
| 10 | 8h | β-actin | 3827.682834 | 4403.649 | 364 | 700 |
| 11 | 11h | β-actin | 1170.767795 | 1746.734 | 364 | 700 |
| 12 | 11h | β-actin | 4175.138889 | 4751.105 | 364 | 700 |
| 13 | 24h | β-actin | 8899.207248 | 9475.174 | 364 | 700 |
| 14 | 24h | β-actin | 7815.426975 | 8391.394 | 364 | 700 |
| 15 | si-YB-1 | β-actin | 9346.043186 | 9922.01 | 364 | 700 |
| 16 | si-Control | β-actin | 2829.378193 | 3158.502 | 208 | 700 |
| **pYB-1^102^ and β-actin** | | | | | | |
| 2 | Background | Background | 0 | 70.42212 | 168 | 800 |
| 32 | 4h | pYB-1^102^ | 44.3727417 | 142.4607 | 234 | 800 |
| 33 | 4h | pYB-1^102^ | 36.38494873 | 134.4729 | 234 | 800 |
| 34 | 6h | pYB-1^102^ | 38.53070068 | 136.6187 | 234 | 800 |
| 35 | 6h | pYB-1^102^ | 37.70135498 | 135.7893 | 234 | 800 |
| 36 | 7h | pYB-1^102^ | 43.86077881 | 141.9487 | 234 | 800 |
| 37 | 7h | pYB-1^102^ | 41.98626709 | 140.0742 | 234 | 800 |
| 38 | 8h | pYB-1^102^ | 49.21551514 | 147.3035 | 234 | 800 |
| 39 | 8h | pYB-1^102^ | 54.0043335 | 152.0923 | 234 | 800 |
| 40 | 11h | pYB-1^102^ | 25.15789795 | 123.2458 | 234 | 800 |
| 41 | 11h | pYB-1^102^ | 27.59173584 | 125.6797 | 234 | 800 |
| 42 | 24h | pYB-1^102^ | 67.78704834 | 165.875 | 234 | 800 |
| 43 | 24h | pYB-1^102^ | 50.04901123 | 148.137 | 234 | 800 |
| 44 | si-YB-1 | pYB-1^102^ | 10.05657959 | 108.1445 | 234 | 800 |
| 45 | si-Control | pYB-1^102^ | 70.43786621 | 206.252 | 324 | 800 |
| 1 | Background | β-actin | 0 | 215.3369 | 144 | 700 |
| 3 | 4h | β-actin | 4362.976318 | 4847.484 | 324 | 700 |
| 17 | 4h | β-actin | 4084.200928 | 4568.709 | 324 | 700 |
| 18 | 6h | β-actin | 4646.887451 | 5131.396 | 324 | 700 |
| 19 | 6h | β-actin | 4301.916748 | 4786.425 | 324 | 700 |
| 20 | 7h | β-actin | 4772.799561 | 5257.308 | 324 | 700 |
| 23 | 7h | β-actin | 5152.042725 | 5636.551 | 324 | 700 |
| 24 | 8h | β-actin | 5979.871826 | 6464.38 | 324 | 700 |
| 25 | 8h | β-actin | 5838.553467 | 6323.062 | 324 | 700 |
| 26 | 11h | β-actin | 4439.948975 | 4924.457 | 324 | 700 |
| 27 | 11h | β-actin | 4513.39624 | 4997.904 | 324 | 700 |
| 28 | 24h | β-actin | 6490.949951 | 6975.458 | 324 | 700 |
| 29 | 24h | β-actin | 4962.108154 | 5446.616 | 324 | 700 |
| 30 | si-YB-1 | β-actin | 6453.905029 | 6938.413 | 324 | 700 |
| 31 | si-Control | β-actin | 8673.224569 | 9256.429 | 390 | 700 |
