# Supplementary material for: Dephosphorylation of YB-1 is Required for Nuclear Localisation During G2 Phase of the Cell Cycle
Source: Cancers (Basel). 2020 Jan 29;12(2):315. doi: 10.3390/cancers12020315 (PMC7072210; doi:10.3390/cancers12020315)
Supplement: Supplementary file 1 [file cancers-12-00315-s001.zip › cancers-661288-v2-suppl/Supplementary Tables/Supplementary Tables of intensity S6&7.docx]

Table S6: Signal intensity for YB-1^HA^, ^FLAG^YB-1 and H3 in A549 whole cell lysates (WCL), nuclear (Nuc) and cytoplasmic (Cyto) fractions.

| **Box Number** | **Sample Description** | **Signal Detected** | **Signal** | **Total** | **Area** | **Channel** |
| --- | --- | --- | --- | --- | --- | --- |
| **YB-1^HA^** | | | | | | |
| 1 | Background | Background | 0 | 320.7178 | 200 | 800 |
| 3 | WCL - DMSO | YB-1^HA^ | 61178.51011 | 62304.231 | 702 | 800 |
| 4 | Nuc - DMSO | YB-1^HA^ | 63466.96616 | 64592.688 | 702 | 800 |
| 5 | Cyto - DMSO | YB-1^HA^ | 5198.497412 | 6324.2168 | 702 | 800 |
| 6 | WCL - PTX | YB-1^HA^ | 48707.06382 | 49832.781 | 702 | 800 |
| 7 | Nuc - PTX | YB-1^HA^ | 45438.37925 | 46564.098 | 702 | 800 |
| 8 | Cyto - PTX | YB-1^HA^ | 13645.79819 | 14771.518 | 702 | 800 |
| 9 | WCL - DOX | YB-1^HA^ | 40701.58042 | 41827.301 | 702 | 800 |
| 10 | Nuc - DOX | YB-1^HA^ | 36525.34214 | 37651.063 | 702 | 800 |
| 11 | Cyto - DOX | YB-1^HA^ | 11212.70347 | 12338.423 | 702 | 800 |
| **^FLAG^YB-1** | | | | | | |
| 2 | Background | Background | 0 | 971.6484 | 192 | 700 |
| 12 | WCL - DMSO | ^FLAG^YB-1 | 25498.99146 | 28768.184 | 646 | 700 |
| 13 | Nuc - DMSO | ^FLAG^YB-1 | 23043.06177 | 26312.254 | 646 | 700 |
| 14 | Cyto - DMSO | ^FLAG^YB-1 | -152.114013 | 3117.078 | 646 | 700 |
| 15 | WCL - PTX | ^FLAG^YB-1 | 17379.41138 | 20648.604 | 646 | 700 |
| 16 | Nuc - PTX | ^FLAG^YB-1 | 13802.40942 | 17071.602 | 646 | 700 |
| 17 | Cyto - PTX | ^FLAG^YB-1 | 1327.487549 | 4596.680 | 646 | 700 |
| 18 | WCL - DOX | ^FLAG^YB-1 | 12617.83521 | 15887.027 | 646 | 700 |
| 19 | Nuc - DOX | ^FLAG^YB-1 | 14964.69458 | 18233.887 | 646 | 700 |
| 20 | Cyto - DOX | ^FLAG^YB-1 | 771.6008301 | 4040.793 | 646 | 700 |
| **H3** | | | | | | |
| 1 | Background | Background | 0 | 125.76392 | 250 | 800 |
| 2 | WCL - DMSO | H3 | 10061.28504 | 10453.667 | 780 | 800 |
| 3 | Nuc - DMSO | H3 | 6130.515996 | 6522.5994 | 780 | 800 |
| 4 | Cyto - DMSO | H3 | 822.4578906 | 1214.8413 | 780 | 800 |
| 5 | WCL - PTX | H3 | 7759.369512 | 8151.7529 | 780 | 800 |
| 6 | Nuc - PTX | H3 | 8885.93543 | 9278.3183 | 780 | 800 |
| 7 | Cyto - PTX | H3 | 1393.07459 | 1785.4580 | 780 | 800 |
| 8 | WCL - DOX | H3 | 4295.902227 | 4688.2857 | 780 | 800 |
| 9 | Nuc - DOX | H3 | 2271.857793 | 2664.2412 | 780 | 800 |
| - | Cyto - DOX | No image to quantify | | | | |

Table S7: Signal intensity for YB-1^HA^, ^FLAG^YB-1 and H3 in H1299 whole cell lysates (WCL), nuclear (Nuc) and cytoplasmic (Cyto) fractions.

| **Box Number** | **Sample Description** | **Signal Detected** | **Signal** | **Total** | **Area** | **Channel** |
| --- | --- | --- | --- | --- | --- | --- |
| **^YB-1HA^** | | | | | | |
| 1 | Background | Background | 0 | 100.2783 | 133 | 800 |
| 3 | WCL - DMSO | YB-1^HA^ | 38116.64886 | 38646.691 | 703 | 800 |
| 12 | Nuc - DMSO | YB-1^HA^ | 40315.06975 | 40845.113 | 703 | 800 |
| 13 | Cyto - DMSO | YB-1^HA^ | 15022.51214 | 15552.554 | 703 | 800 |
| 14 | WCL - PTX | YB-1^HA^ | 30088.63955 | 30618.681 | 703 | 800 |
| 15 | Nuc - PTX | YB-1^HA^ | 34255.73345 | 34785.777 | 703 | 800 |
| 16 | Cyto - PTX | YB-1^HA^ | 12052.32903 | 12582.371 | 703 | 800 |
| 17 | WCL - DOX | YB-1^HA^ | 23677.0114 | 24207.055 | 703 | 800 |
| 18 | Nuc - DOX | YB-1^HA^ | 19174.14446 | 19704.188 | 703 | 800 |
| 19 | Cyto - DOX | YB-1^HA^ | 21098.72781 | 21628.769 | 703 | 800 |
| **YB-1^FLAG^** | | | | | | |
| 2 | Background | Background | 0 | 483.375 | 112 | 700 |
| 20 | WCL - DMSO | ^FLAG^YB-1 | 17670.60435 | 20864.332 | 740 | 700 |
| 29 | Nuc - DMSO | ^FLAG^YB-1 | 18823.5106 | 22017.238 | 740 | 700 |
| 30 | Cyto - DMSO | ^FLAG^YB-1 | 4582.741071 | 7776.468 | 740 | 700 |
| 31 | WCL - PTX | ^FLAG^YB-1 | 13753.78404 | 16947.511 | 740 | 700 |
| 32 | Nuc - PTX | ^FLAG^YB-1 | 15923.67857 | 19117.406 | 740 | 700 |
| 33 | Cyto - PTX | ^FLAG^YB-1 | 2894.399275 | 6088.127 | 740 | 700 |
| 34 | WCL - DOX | ^FLAG^YB-1 | 12885.29381 | 16079.021 | 740 | 700 |
| 35 | Nuc - DOX | ^FLAG^YB-1 | 11527.06724 | 14720.795 | 740 | 700 |
| 36 | Cyto - DOX | ^FLAG^YB-1 | 9046.895368 | 12240.623 | 740 | 700 |
| **H3** | | | | | | |
| 1 | Background | Background | 0 | 51.345703 | 100 | 800 |
| 2 | WCL - DMSO | H3 | 2240.729375 | 2461.0024 | 429 | 800 |
| 3 | Nuc - DMSO | H3 | 354.3123828 | 574.58544 | 429 | 800 |
| 4 | Cyto - DMSO | H3 | 75.22546875 | 295.49853 | 429 | 800 |
| 5 | WCL - PTX | H3 | 276.0799609 | 496.35302 | 429 | 800 |
| 6 | Nuc - PTX | H3 | 230.354375 | 450.62744 | 429 | 800 |
| 7 | Cyto - PTX | H3 | 22.39758789 | 242.67065 | 429 | 800 |
| 8 | WCL - DOX | H3 | 323.2723438 | 543.54541 | 429 | 800 |
| 9 | Nuc - DOX | H3 | 291.3460742 | 511.61914 | 429 | 800 |
| 10 | Cyto - DOX | H3 | 64.23914063 | 284.51221 | 429 | 800 |
