# Supplementary material for: Dephosphorylation of YB-1 is Required for Nuclear Localisation During G2 Phase of the Cell Cycle
Source: Cancers (Basel). 2020 Jan 29;12(2):315. doi: 10.3390/cancers12020315 (PMC7072210; doi:10.3390/cancers12020315)
Supplement: Supplementary file 1 [file cancers-12-00315-s001.zip › cancers-661288-v2-suppl/Supplementary Tables/Table S8_primer sequences.docx]

**Table S8. Primer sequences used for generation of *^HA^*YB-1*^FLAG^* and *^FLAG^*YB-1*^HA^* expression constructs**

| S102A Fwd | 5'-ccaggaagtaccttcgcgctgtaggagatggagaga-3' |
| --- | --- |
| S102A Rev | 5'-tctctccatctcctacagcgcgaaggtacttcctgg-3' |
| S165A Fwd | 5'- cgcaattaccagcaaaattaccagaatgctgagagtggggaaa-3' |
| S165A Rev | 5'- tttccccactctcagcattctggtaattttgctggtaattgcg-3' |
| S176A Fwd | 5'-cgagggatcggaggctgctcccgaaggc-3' |
| S176A Rev | 5'-gccttcgggagcagcctccgatccctcg-3' |
