# Supplementary figures and images for: Dephosphorylation of YB-1 is Required for Nuclear Localisation During G2 Phase of the Cell Cycle
Source: Cancers (Basel). 2020 Jan 29;12(2):315. doi: 10.3390/cancers12020315 (PMC7072210; doi:10.3390/cancers12020315)

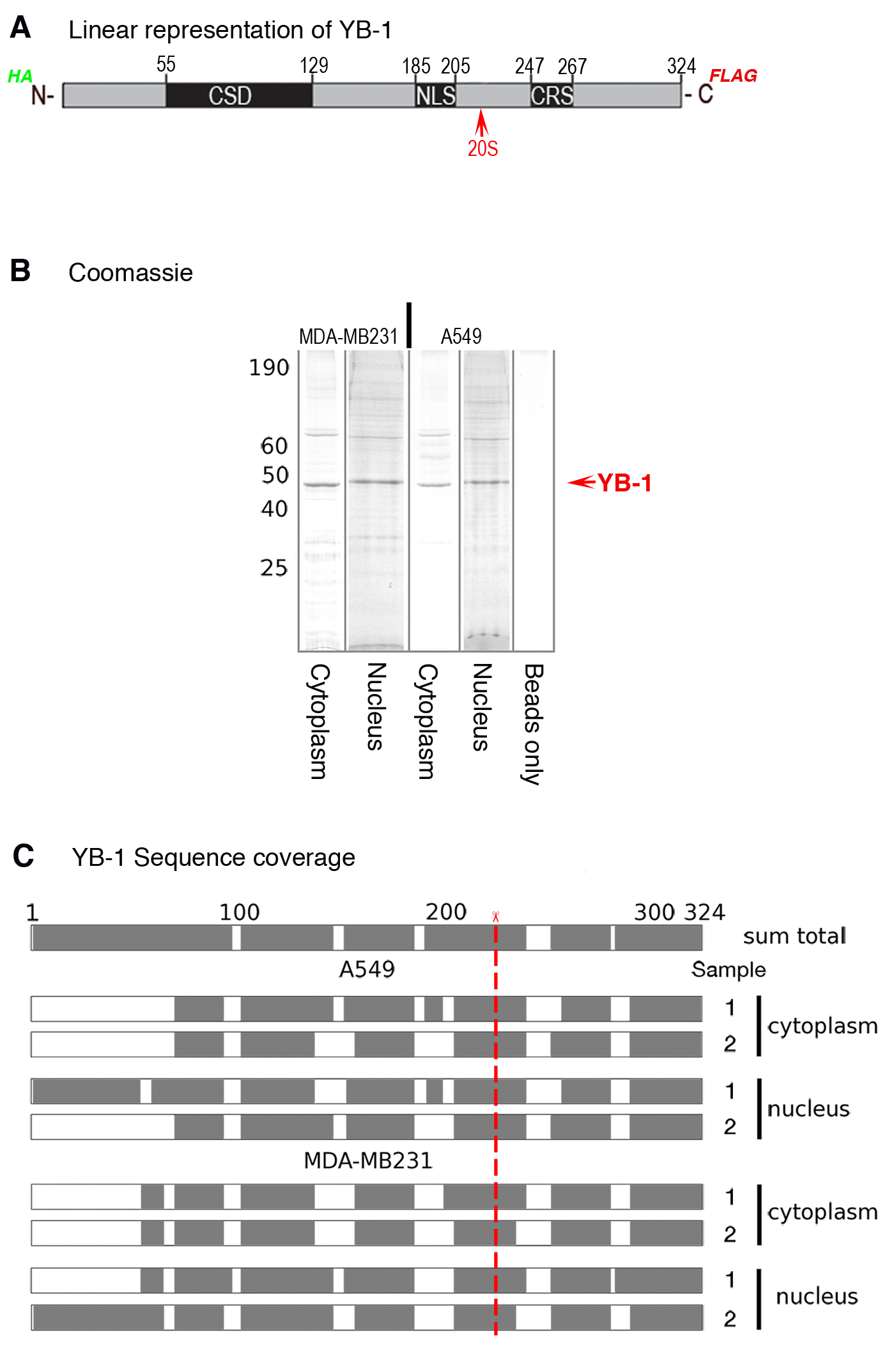

Supplement: Supplementary file 1 [file cancers-12-00315-s001.zip › cancers-661288-v2-suppl/Supplementary Figures/Figure S1.tif]

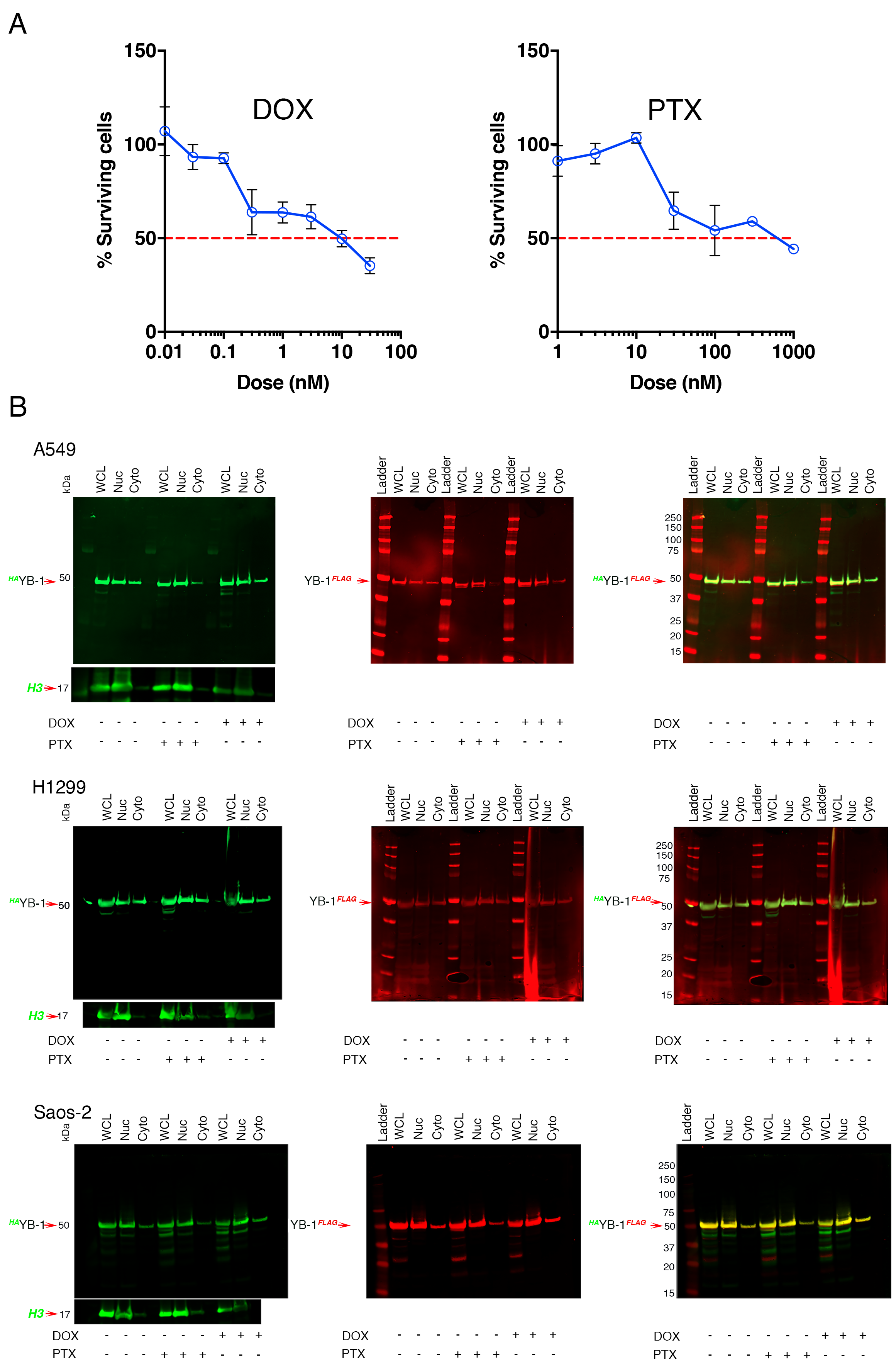

Supplement: Supplementary file 1 [file cancers-12-00315-s001.zip › cancers-661288-v2-suppl/Supplementary Figures/Figure S2.tif]

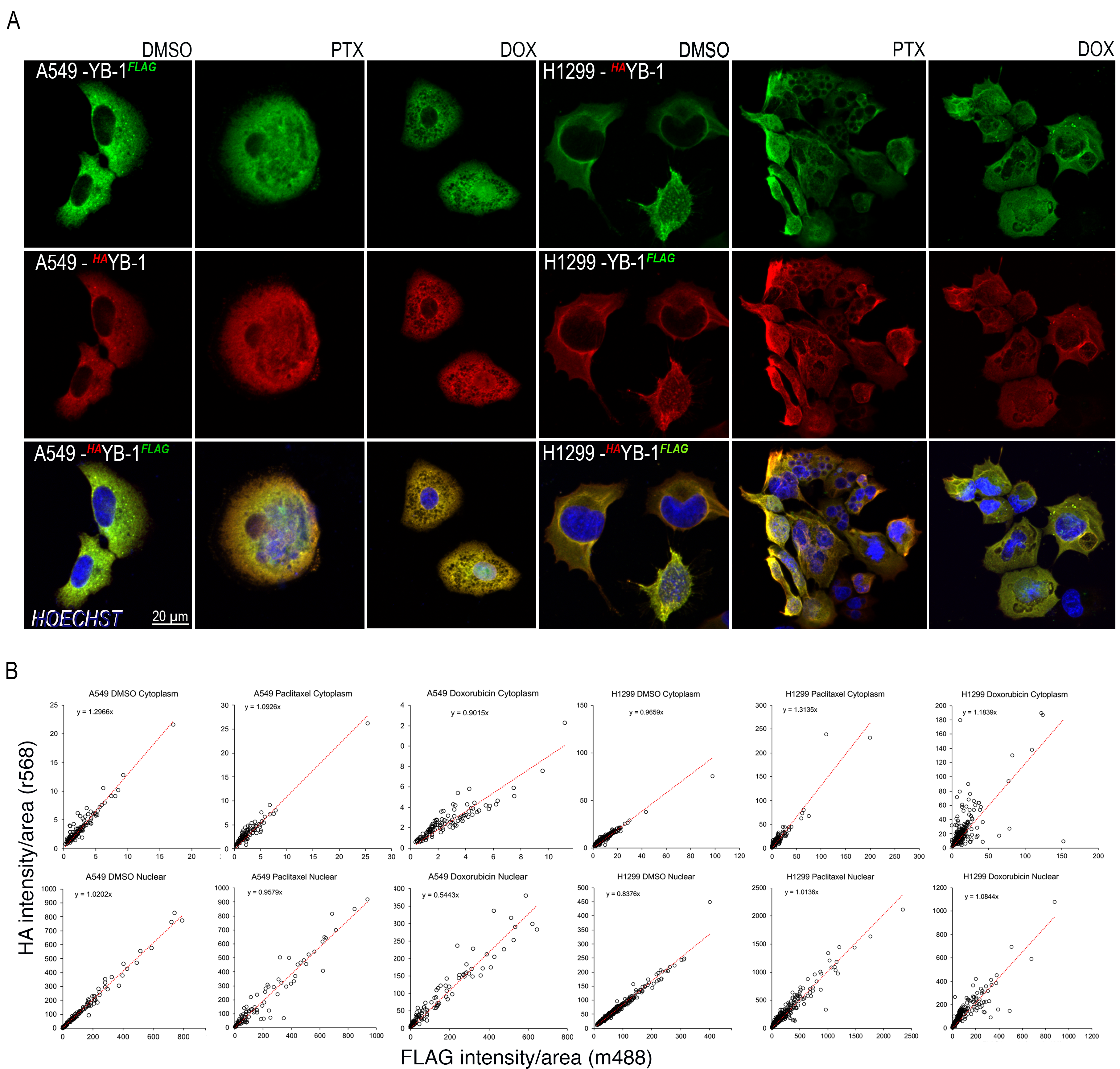

Supplement: Supplementary file 1 [file cancers-12-00315-s001.zip › cancers-661288-v2-suppl/Supplementary Figures/Figure S3.tif]

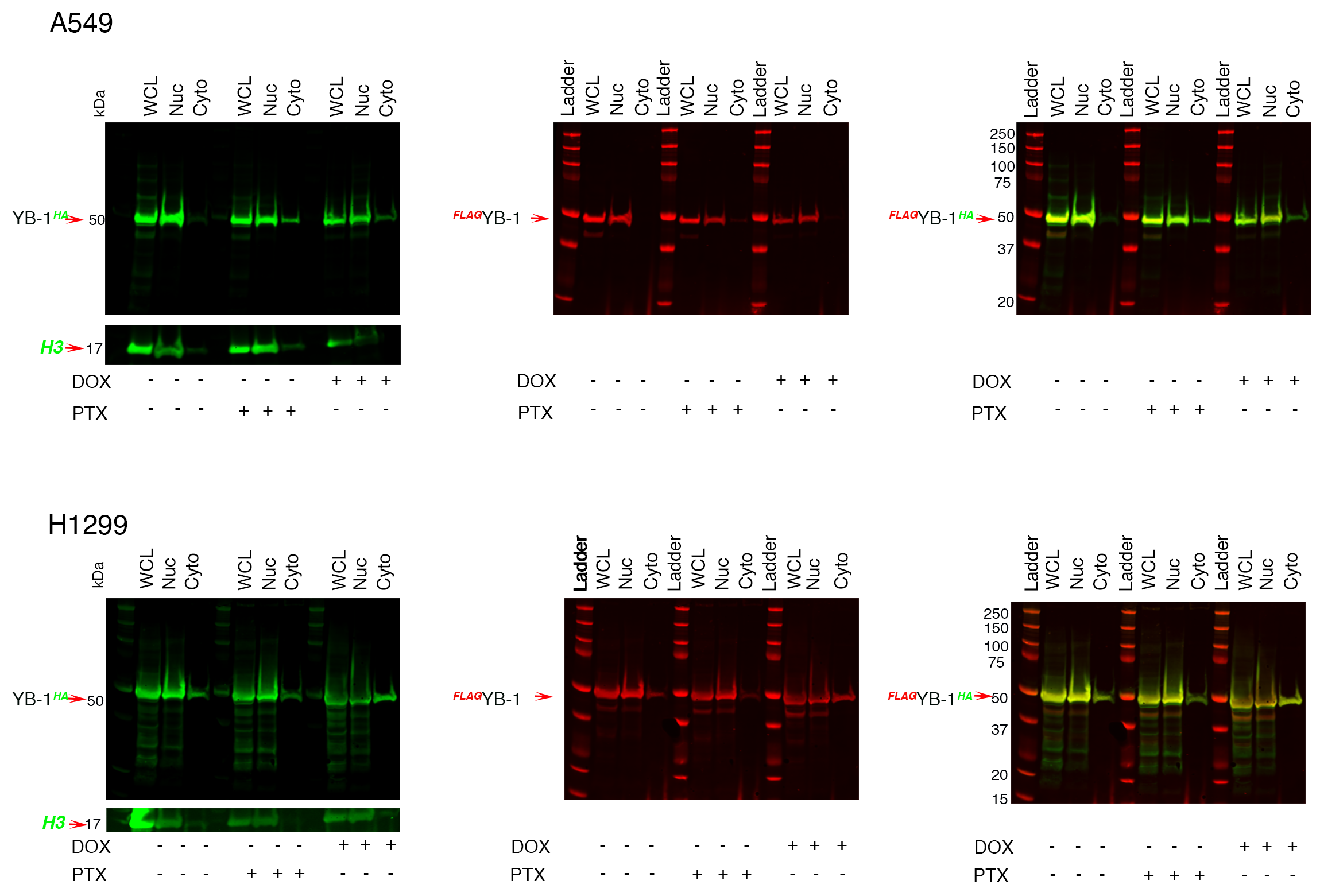

Supplement: Supplementary file 1 [file cancers-12-00315-s001.zip › cancers-661288-v2-suppl/Supplementary Figures/Figure S4.tif]

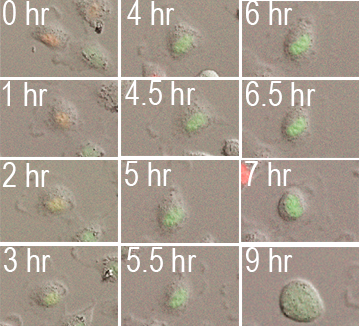

Supplement: Supplementary file 1 [file cancers-12-00315-s001.zip › cancers-661288-v2-suppl/Supplementary Figures/Figure S5.tif]

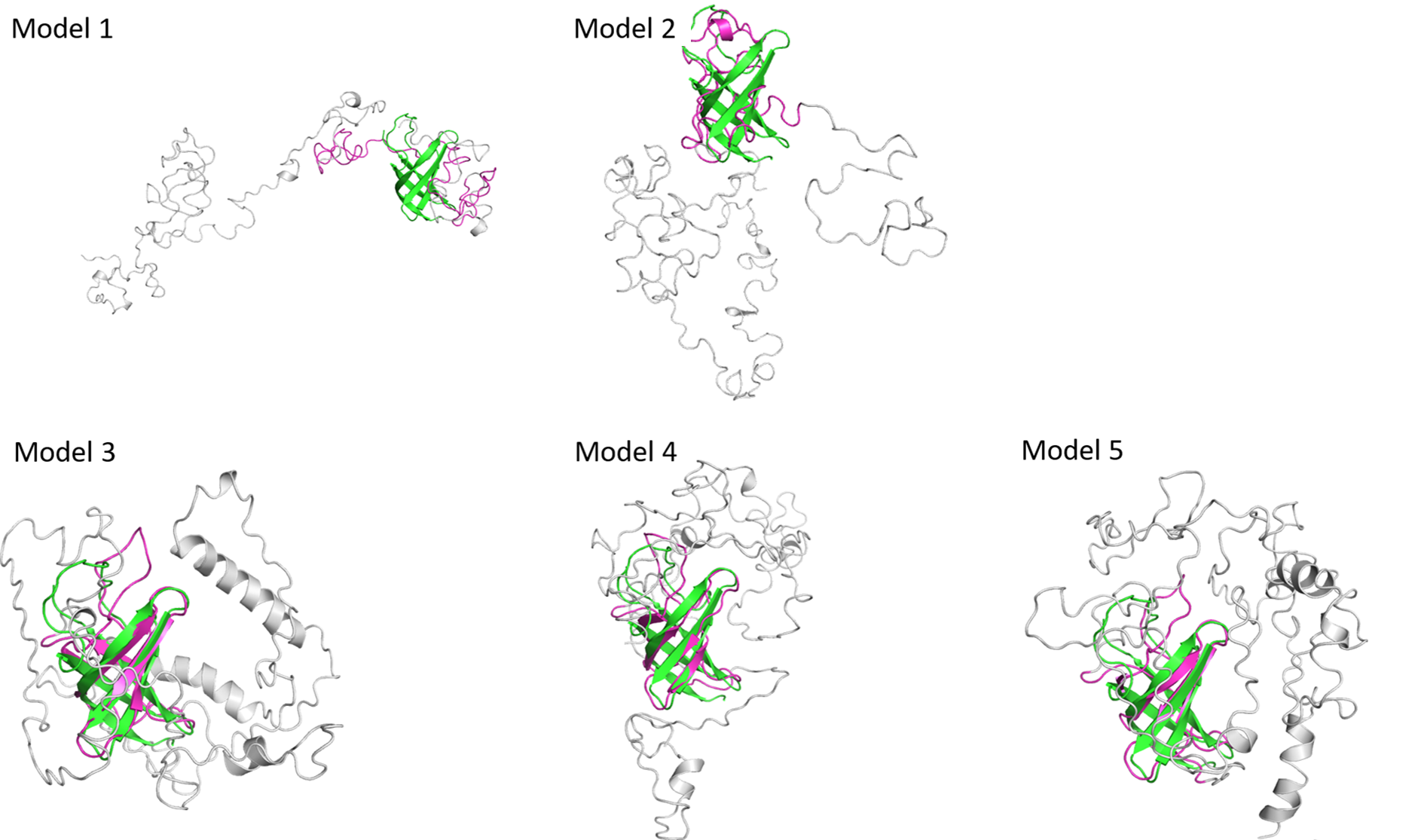

Supplement: Supplementary file 1 [file cancers-12-00315-s001.zip › cancers-661288-v2-suppl/Supplementary Figures/Figure S6.tif]

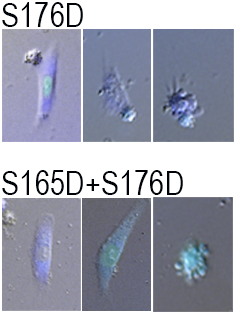

Supplement: Supplementary file 1 [file cancers-12-00315-s001.zip › cancers-661288-v2-suppl/Supplementary Figures/Figure S7.tif]

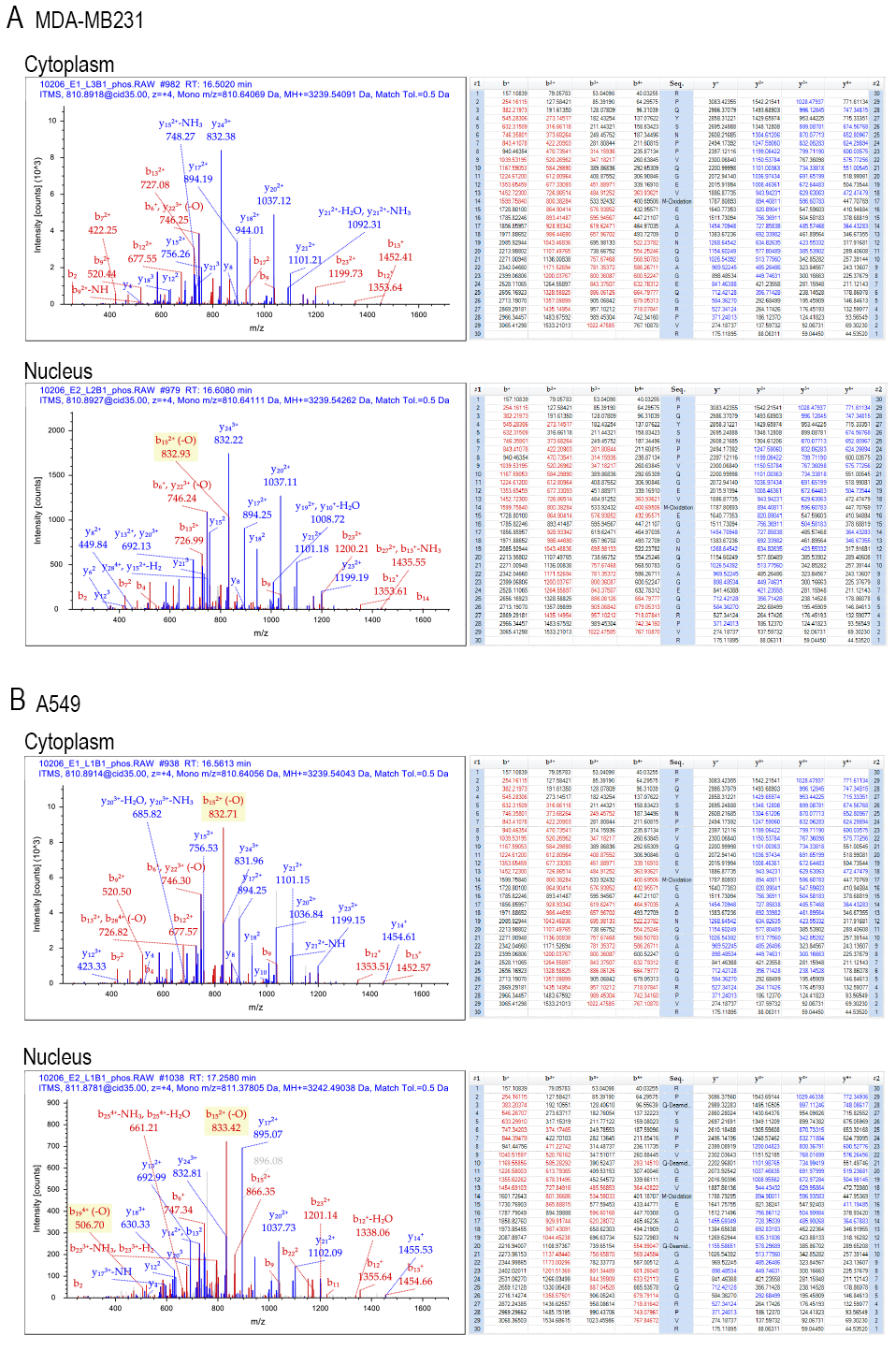

Supplement: Supplementary file 1 [file cancers-12-00315-s001.zip › cancers-661288-v2-suppl/Supplementary Figures/Figure S8.tif]

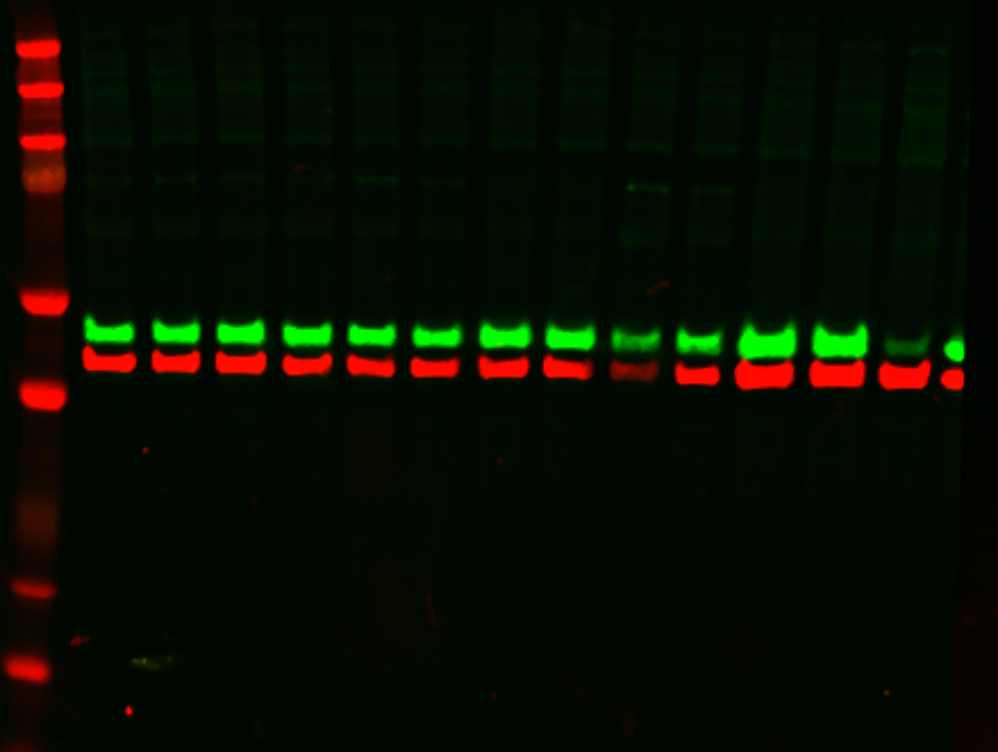

Supplement: Supplementary file 1 [file cancers-12-00315-s001.zip › cancers-661288-v2-suppl/Western blots for Figures 6, S2 and S4_/Full Western Blots for Figure 6/A_Total YB1 & actin.tif]

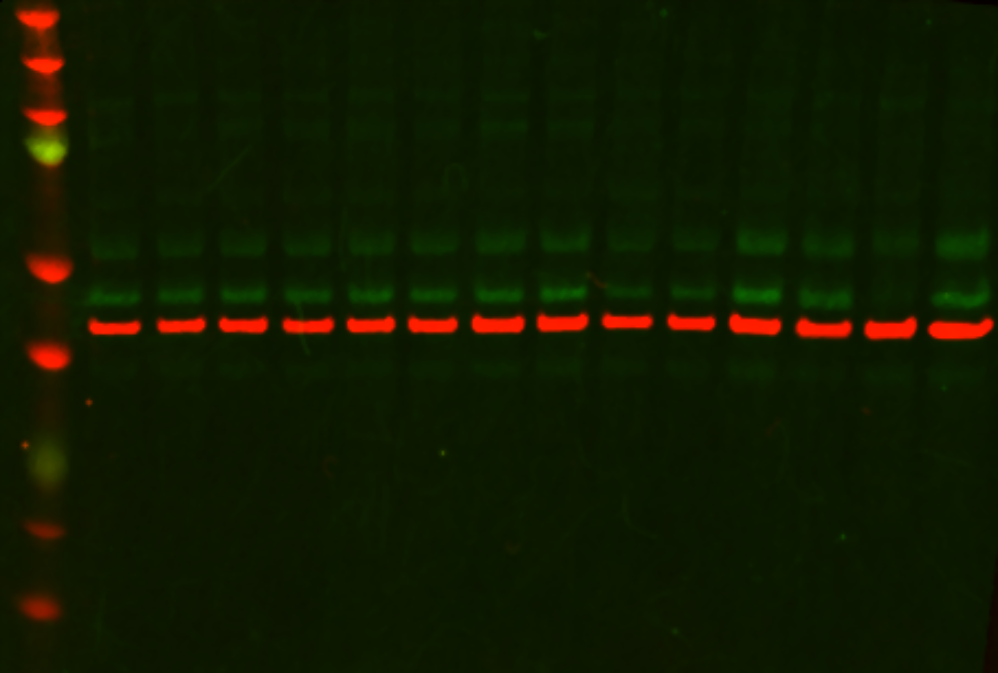

Supplement: Supplementary file 1 [file cancers-12-00315-s001.zip › cancers-661288-v2-suppl/Western blots for Figures 6, S2 and S4_/Full Western Blots for Figure 6/B_YB-1_S102 & actin.tif]

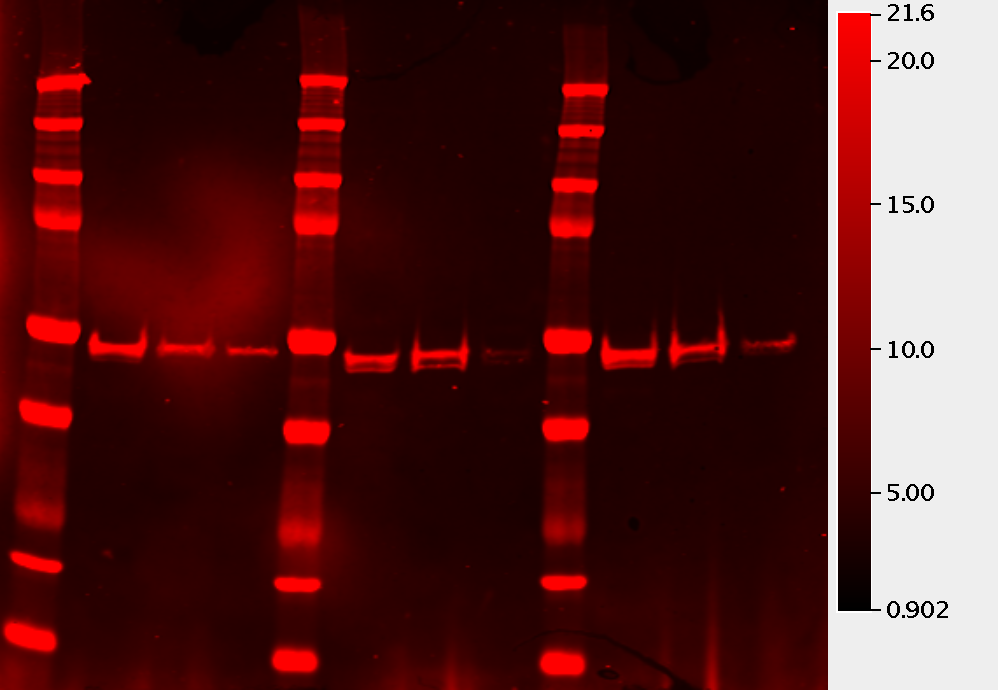

Supplement: Supplementary file 1 [file cancers-12-00315-s001.zip › cancers-661288-v2-suppl/Western blots for Figures 6, S2 and S4_/Full Western Blots for Figure S2/A549 Flag.tif]

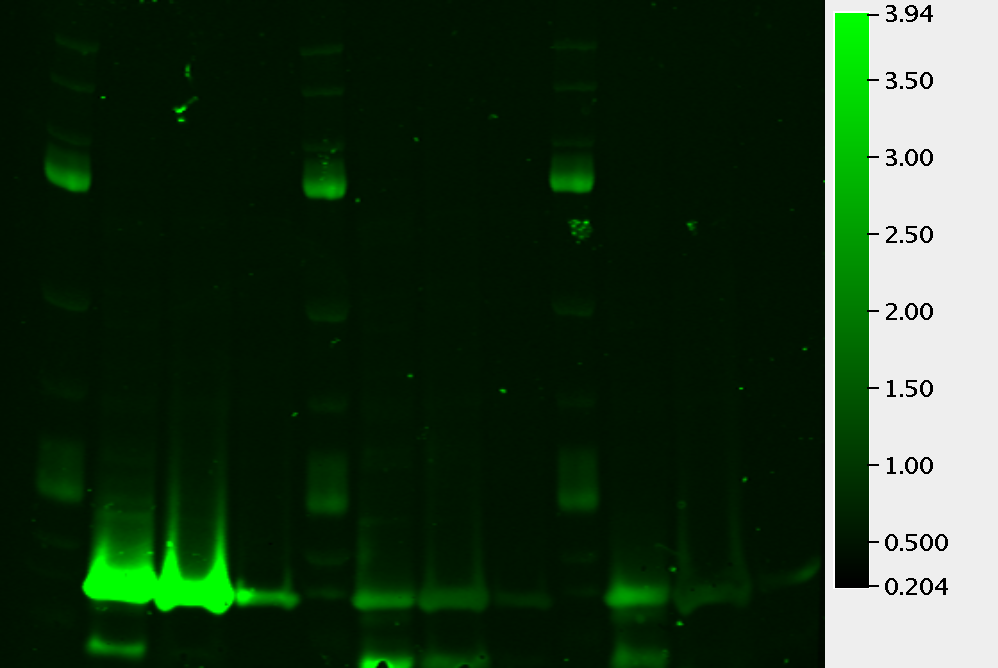

Supplement: Supplementary file 1 [file cancers-12-00315-s001.zip › cancers-661288-v2-suppl/Western blots for Figures 6, S2 and S4_/Full Western Blots for Figure S2/A549 H3.tif]

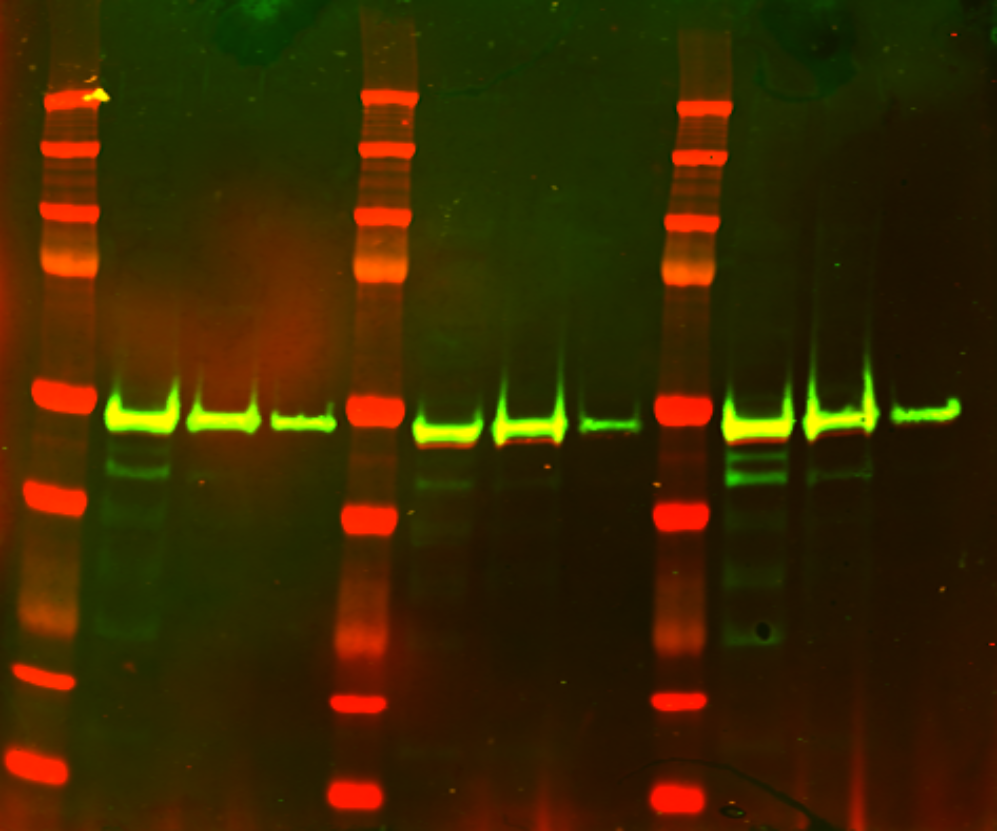

Supplement: Supplementary file 1 [file cancers-12-00315-s001.zip › cancers-661288-v2-suppl/Western blots for Figures 6, S2 and S4_/Full Western Blots for Figure S2/A549 Ha and Flag merge.tif]

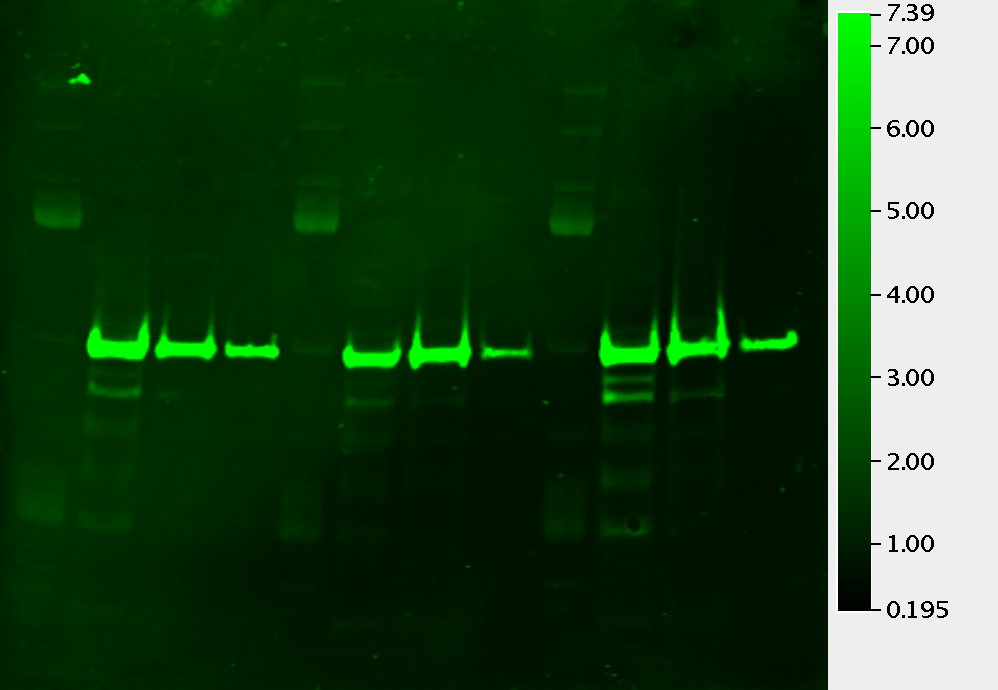

Supplement: Supplementary file 1 [file cancers-12-00315-s001.zip › cancers-661288-v2-suppl/Western blots for Figures 6, S2 and S4_/Full Western Blots for Figure S2/A549 HA.tif]

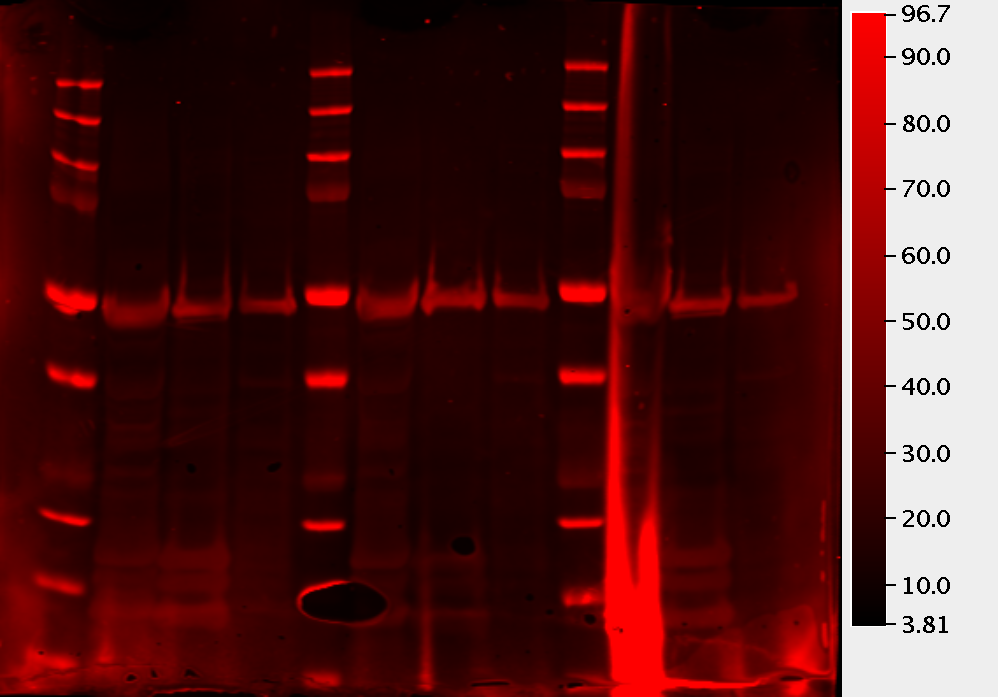

Supplement: Supplementary file 1 [file cancers-12-00315-s001.zip › cancers-661288-v2-suppl/Western blots for Figures 6, S2 and S4_/Full Western Blots for Figure S2/H1299 Flag.tif]

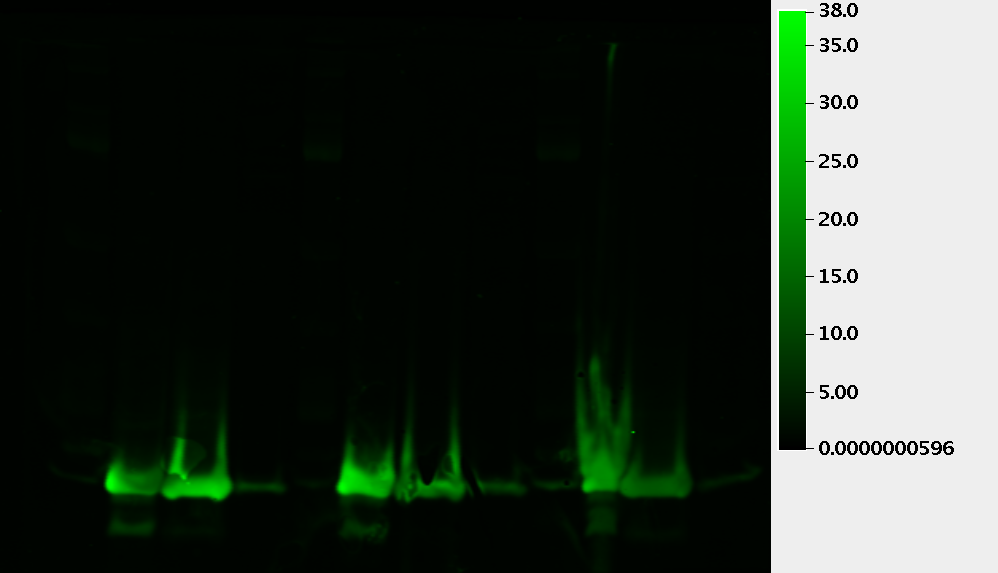

Supplement: Supplementary file 1 [file cancers-12-00315-s001.zip › cancers-661288-v2-suppl/Western blots for Figures 6, S2 and S4_/Full Western Blots for Figure S2/H1299 H3.tif]

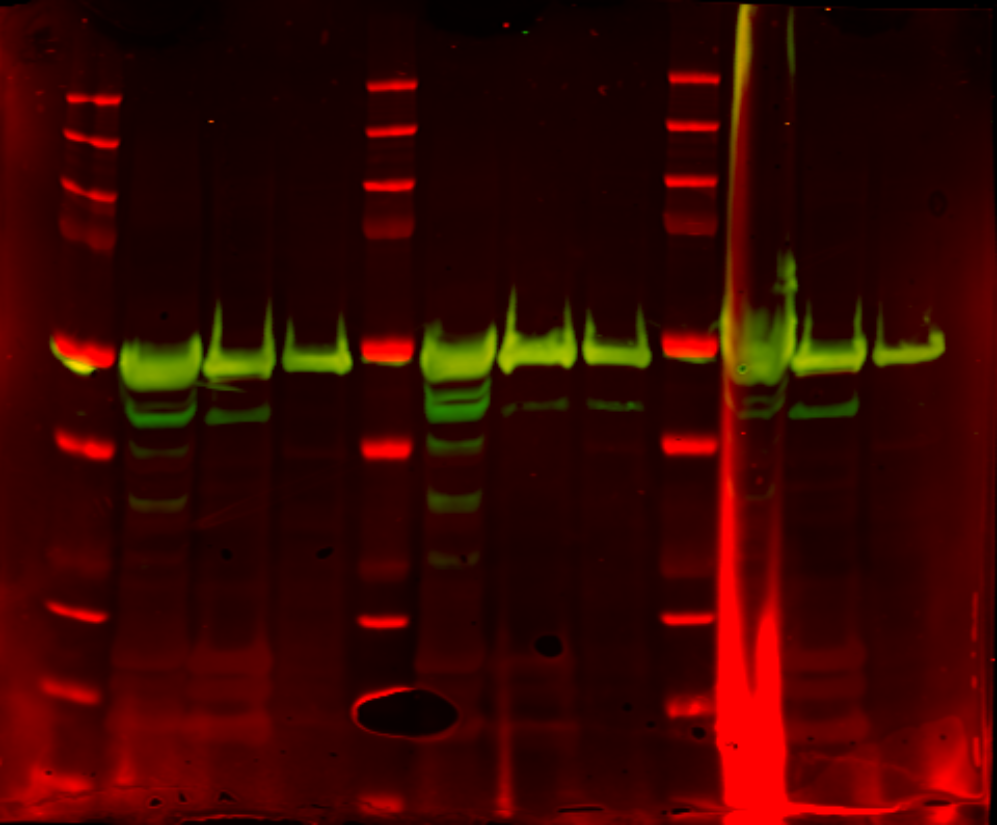

Supplement: Supplementary file 1 [file cancers-12-00315-s001.zip › cancers-661288-v2-suppl/Western blots for Figures 6, S2 and S4_/Full Western Blots for Figure S2/H1299 HA and Flag merge.tif]

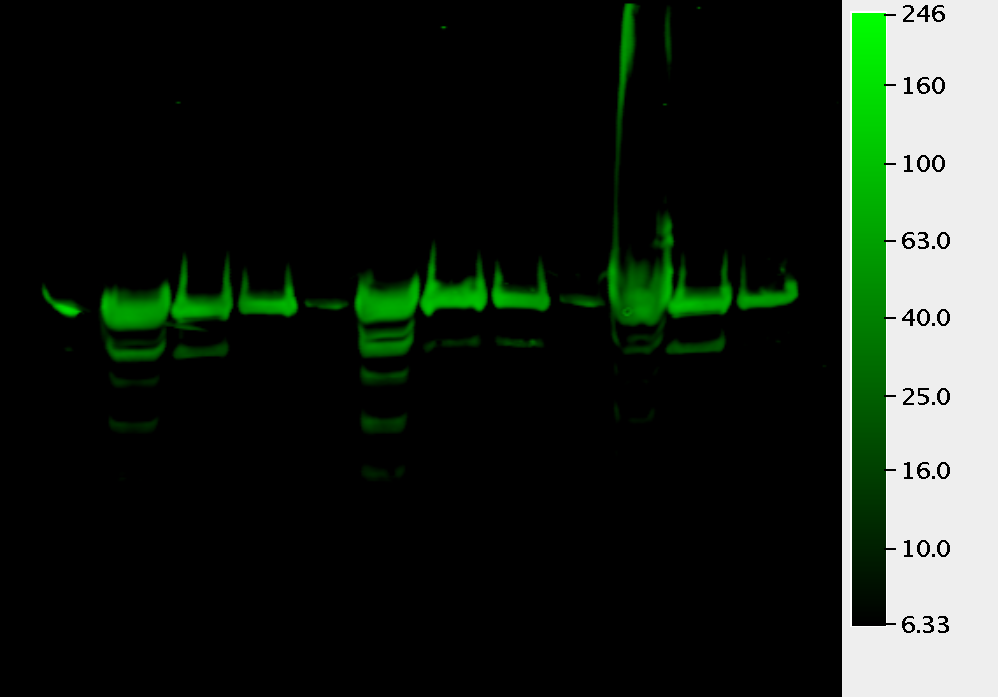

Supplement: Supplementary file 1 [file cancers-12-00315-s001.zip › cancers-661288-v2-suppl/Western blots for Figures 6, S2 and S4_/Full Western Blots for Figure S2/H1299 HA.tif]

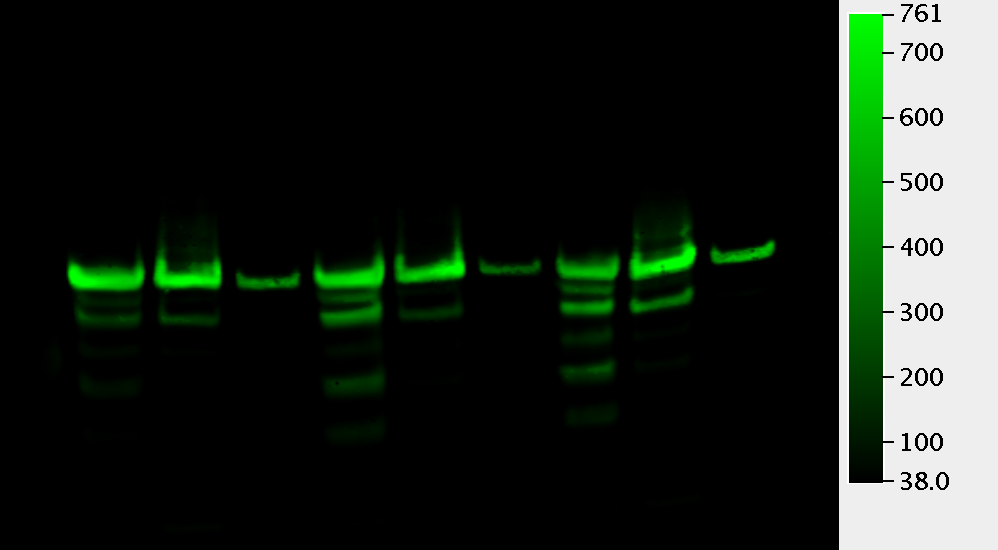

Supplement: Supplementary file 1 [file cancers-12-00315-s001.zip › cancers-661288-v2-suppl/Western blots for Figures 6, S2 and S4_/Full Western Blots for Figure S2/SAOS Flag.tif]

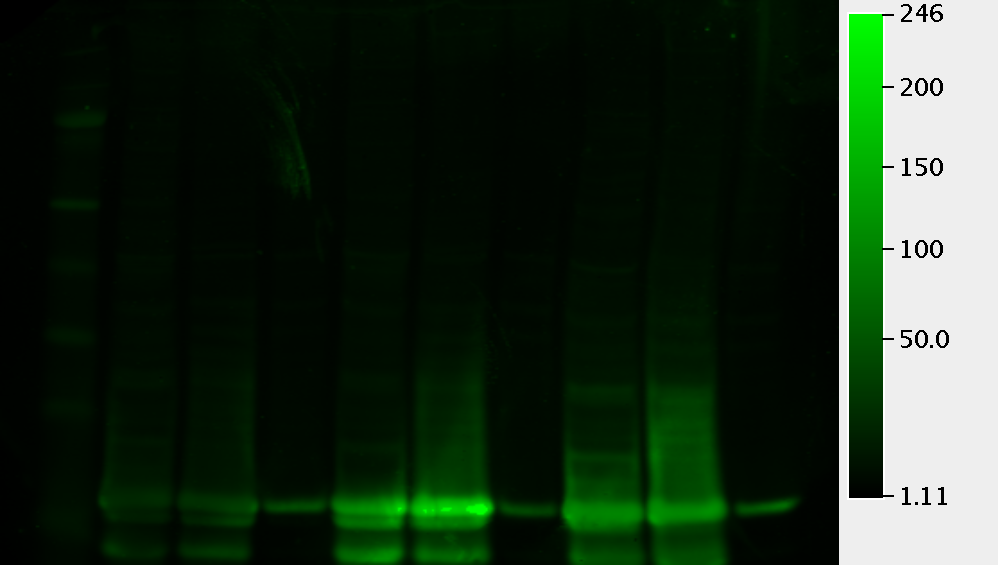

Supplement: Supplementary file 1 [file cancers-12-00315-s001.zip › cancers-661288-v2-suppl/Western blots for Figures 6, S2 and S4_/Full Western Blots for Figure S2/SAOS H3.tif]

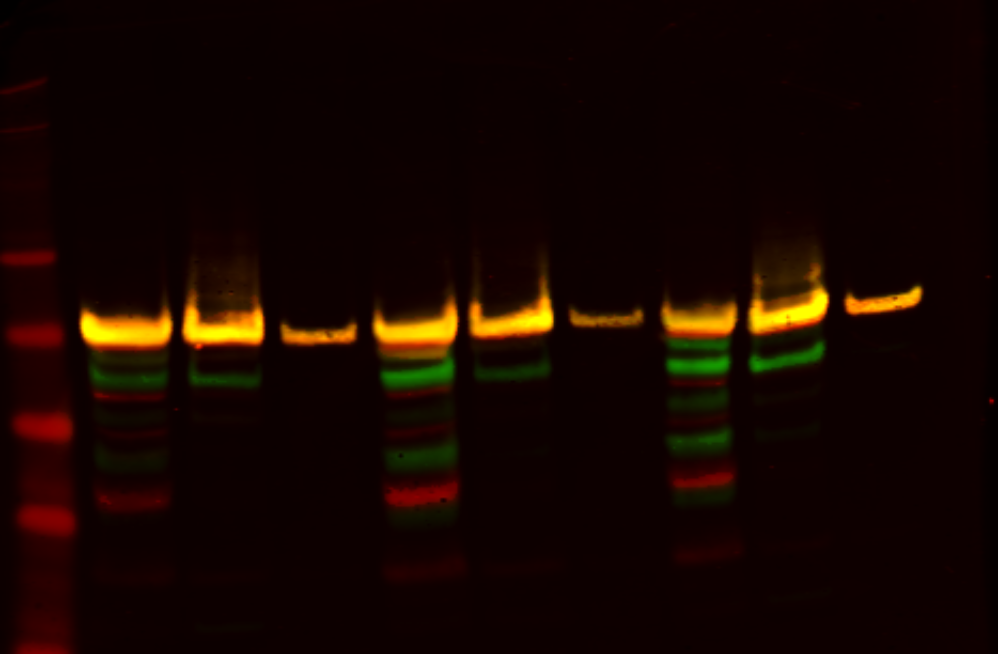

Supplement: Supplementary file 1 [file cancers-12-00315-s001.zip › cancers-661288-v2-suppl/Western blots for Figures 6, S2 and S4_/Full Western Blots for Figure S2/SAOS HA Flag merge.tif]

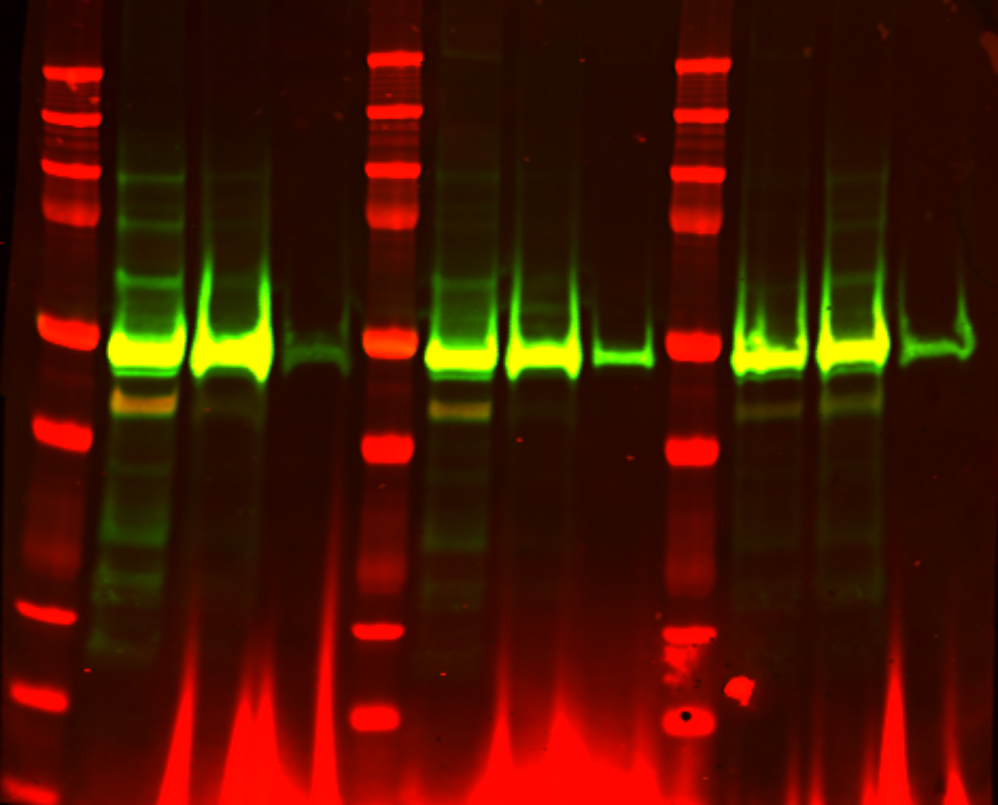

Supplement: Supplementary file 1 [file cancers-12-00315-s001.zip › cancers-661288-v2-suppl/Western blots for Figures 6, S2 and S4_/Full Western blots for Figure S4/A549 FYH FLAG and HA merge.tif]

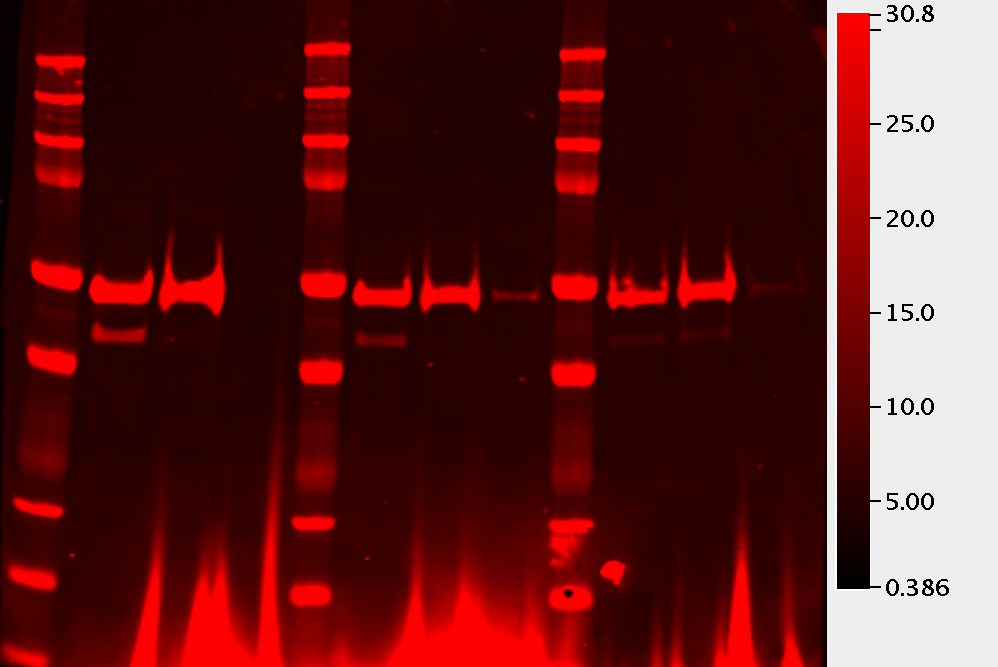

Supplement: Supplementary file 1 [file cancers-12-00315-s001.zip › cancers-661288-v2-suppl/Western blots for Figures 6, S2 and S4_/Full Western blots for Figure S4/A549 FYH FLAG.tif]

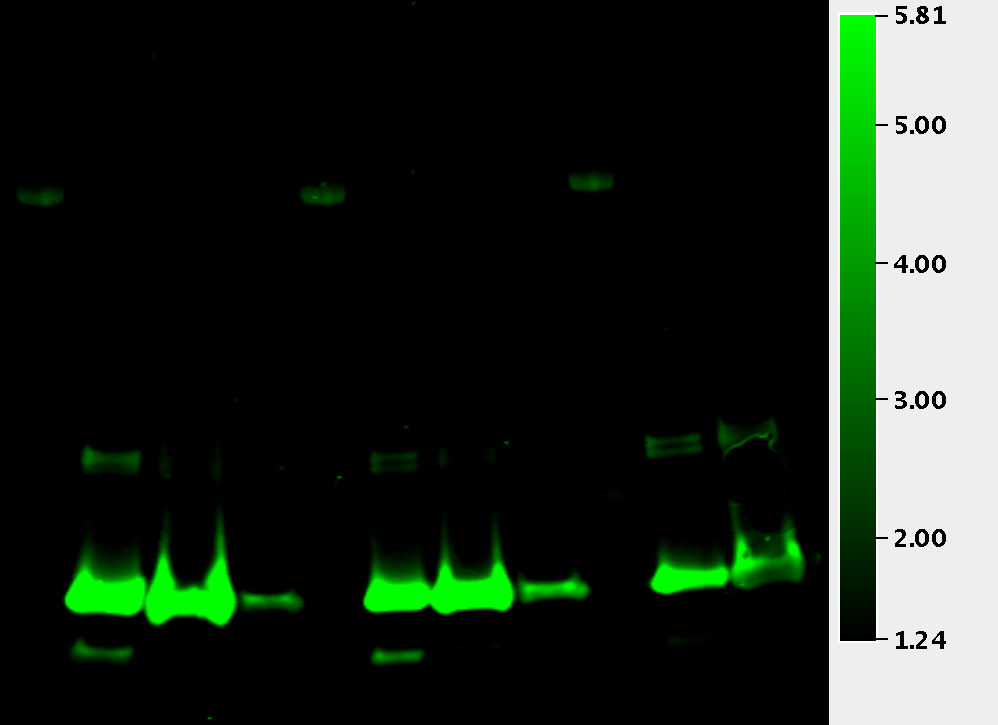

Supplement: Supplementary file 1 [file cancers-12-00315-s001.zip › cancers-661288-v2-suppl/Western blots for Figures 6, S2 and S4_/Full Western blots for Figure S4/A549 FYH H3.tif]

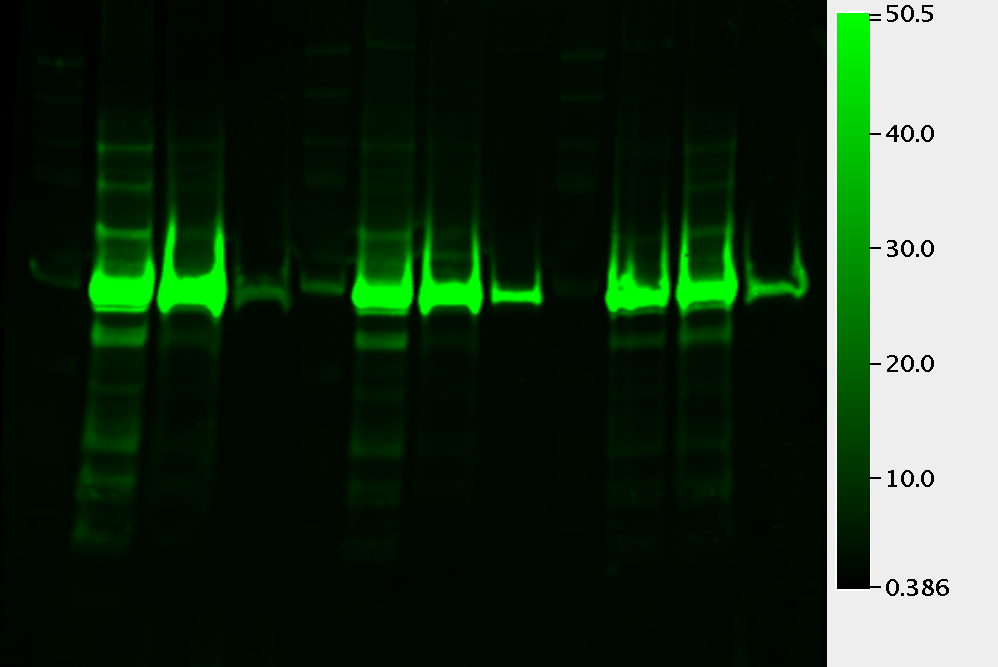

Supplement: Supplementary file 1 [file cancers-12-00315-s001.zip › cancers-661288-v2-suppl/Western blots for Figures 6, S2 and S4_/Full Western blots for Figure S4/A549 FYH HA.tif]

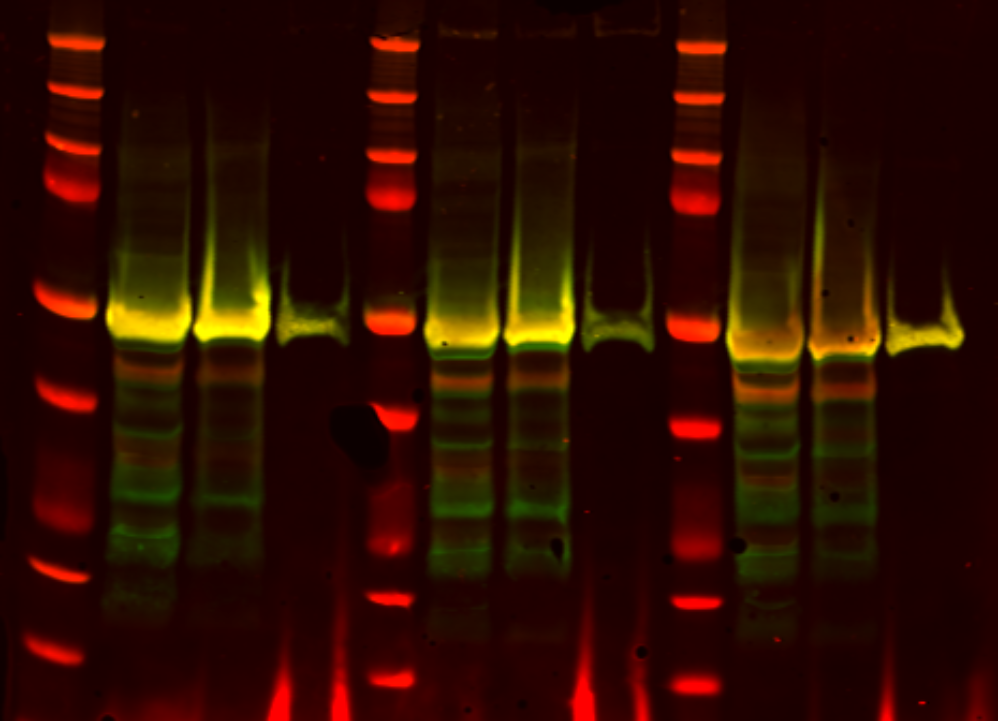

Supplement: Supplementary file 1 [file cancers-12-00315-s001.zip › cancers-661288-v2-suppl/Western blots for Figures 6, S2 and S4_/Full Western blots for Figure S4/H1299 FYH FLAG and HA merge.tif]

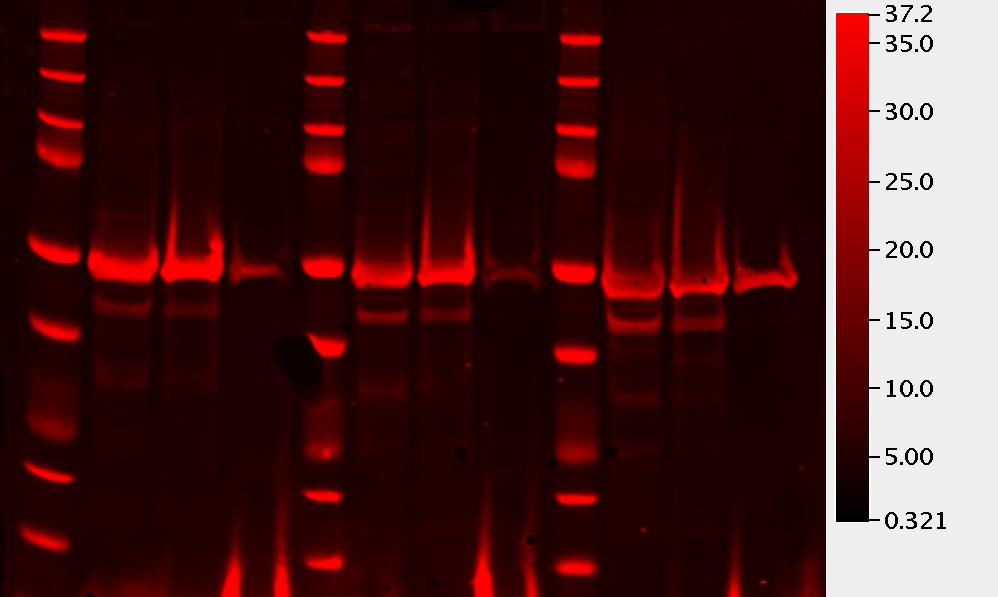

Supplement: Supplementary file 1 [file cancers-12-00315-s001.zip › cancers-661288-v2-suppl/Western blots for Figures 6, S2 and S4_/Full Western blots for Figure S4/H1299 FYH FLAG.tif]

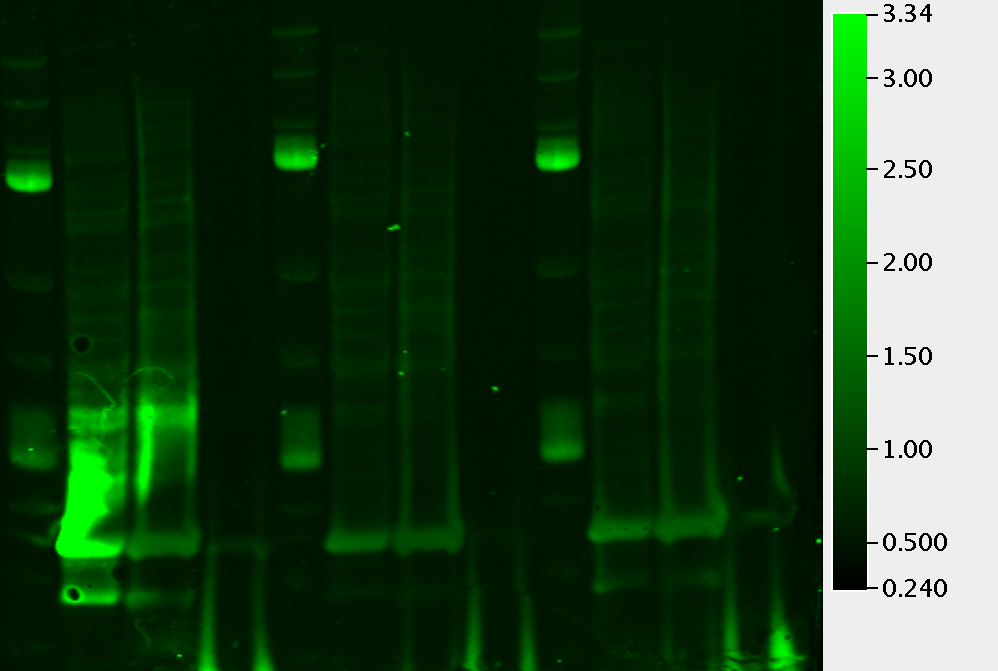

Supplement: Supplementary file 1 [file cancers-12-00315-s001.zip › cancers-661288-v2-suppl/Western blots for Figures 6, S2 and S4_/Full Western blots for Figure S4/H1299 FYH H3.tif]

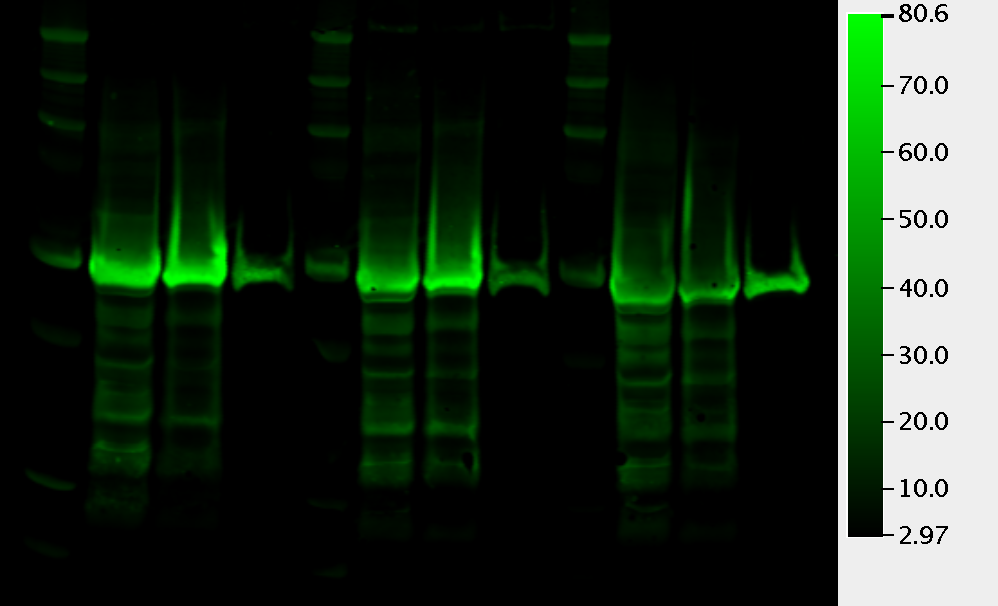

Supplement: Supplementary file 1 [file cancers-12-00315-s001.zip › cancers-661288-v2-suppl/Western blots for Figures 6, S2 and S4_/Full Western blots for Figure S4/H1299 FYH HA.tif]
